# Detection of monkeypox virus using helicase dependent amplification and recombinase polymerase amplification combined with lateral flow test

**DOI:** 10.1186/s12985-023-02223-8

**Published:** 2023-11-23

**Authors:** Abudushalamu Gulinaizhaer, Chuankun Yang, Mingyuan Zou, Shuo Ma, Xiaobo Fan, Guoqiu Wu

**Affiliations:** 1 Zhongda Hospital, Center of Clinical Laboratory Medicine, Medical School, Southeast University, Nanjing, 210009 People’s Republic of China; 2 Diagnostics Department, Medical School of Southeast University, Nanjing, 210009 People’s Republic of China; 3 Jiangsu Provincial Key Laboratory of Critical Care Medicine, Southeast University, Nanjing, 210009 People’s Republic of China

**Keywords:** Monkeypox, INAAT, HDA, RPA, LFT, qPCR

## Abstract

The monkeypox virus (MPXV) is a zoonotic DNA virus that belongs to the poxvirus family. Conventional laboratory methods for detecting MPXV are complex and expensive, making them unsuitable for detecting the virus in regions with limited resources. In this study, we using the Helicase dependent amplification (HDA) method and the Recombinase polymerase amplification (RPA) technique in combination with the lateral flow test (LFT), together with a self-designed qPCR technique for the detection of the MPXV specific conserved fragment F3L, to compare the sensitivity and specificity of the three assays. By analyzing the sensitivity detection results using Probit, it can be seen that the limit of detection (LOD) of the HDA-LFT detection target is 9.86 copies/µL (95% confidence interval, CI 7.52 copies/µL lower bound), the RPA-LFT detection target is 6.97 copies/µL (95% CI 3.90 copies/µL lower bound), and the qPCR detection target is 479.24 copies/mL (95% CI 273.81 copies/mL lower bound). The specificity test results showed that the specificity of the three methods mentioned above was higher than 90% in detecting pseudoviruses of the same genus of MPXV. The simple, highly sensitive, and specific MPXV assay developed in this study is anticipated to provide a solid foundation for future applications in the early screening, diagnosis, and evaluation of the efficacy of MPXV. This is the first time the HDA-LFT assay has been utilized to detect MPXV infection.

**Supplementary Information:**

The online version contains supplementary material available at 10.1186/s12985-023-02223-8.

## Introduction

Monkeypox (MPX) is a zoonotic disease caused by the monkeypox virus (MPXV). MPXV consists of double-stranded DNA and an outer membrane and is a member of the genus *Orthopoxvirus (OPXV)* of the family *Poxviridae* (which includes cowpox virus, smallpox virus, and other pox viruses) [1]. MPXV first originated from the rainforests of the Congo in Central Africa; subsequently, it spread to West Africa over the course of decades of intermittent outbreaks, resulting in West African and Congo Basin strains. The West African strain is significantly less lethal and dangerous than the Congo Basin strain [2]. In addition to being transmitted by monkeys, the virus is also transmitted by rodents such as Gambian kangaroos and African squirrels. MPXV is also capable of infecting humans, but its infection rate and mortality rate are significantly lower than those of smallpox virus. MPXV is primarily transmitted through direct contact with blood, diseased tissues, or large respiratory droplets from infected individuals. Similar to vesicular rash, patients experience headaches, fever, swollen lymph nodes, and other symptoms [3]. The incubation period is typically between 7 and 14 days, and the duration of symptoms is typically between 2 and 4 weeks. In addition, the lethality rate ranges between 0% and 11%, and the vast majority of patients recover [4]. There are currently no targeted treatment options for the MPX epidemic, which has emerged as a worldwide issue; thus, accurate testing is essential for early identification of cases, rapid differential diagnosis, and control of the spread of the virus.

MPXV can be detected using viral isolation and culture techniques, as well as electron microscopic biopsy, immunohistochemical staining for poxvirus antigens, and serological studies for anti-orthopoxvirus *IgM* and *IgG* antibodies. However, the above diagnostic tests are either inconclusive or require lengthy culture procedures. Based on the epidemiological characteristics of the virus and the need for surveillance, the confirmation of MPXV infection is best suited to the rapid detection of MPXV DNA by molecular amplification of viral nucleic acids, specifically by polymerase chain reaction (PCR) techniques [5]. Various diagnostic specimens are obtained directly from the rash, including skin, fluid, and scabs. This permits a rapid and specific early screening diagnosis of the pox virus in a short amount of time. Although PCR is the gold standard for detecting MPXV, the above conditions cannot be met in more remote locations due to the high requirements for operating instruments and operators. Thus, for the early screening, diagnosis, and efficacy evaluation of MPXV, it is crucial to develop a detection method that is easy to operate, convenient and efficient, and sensitive with a high degree of specificity.

**Fig. 1:**
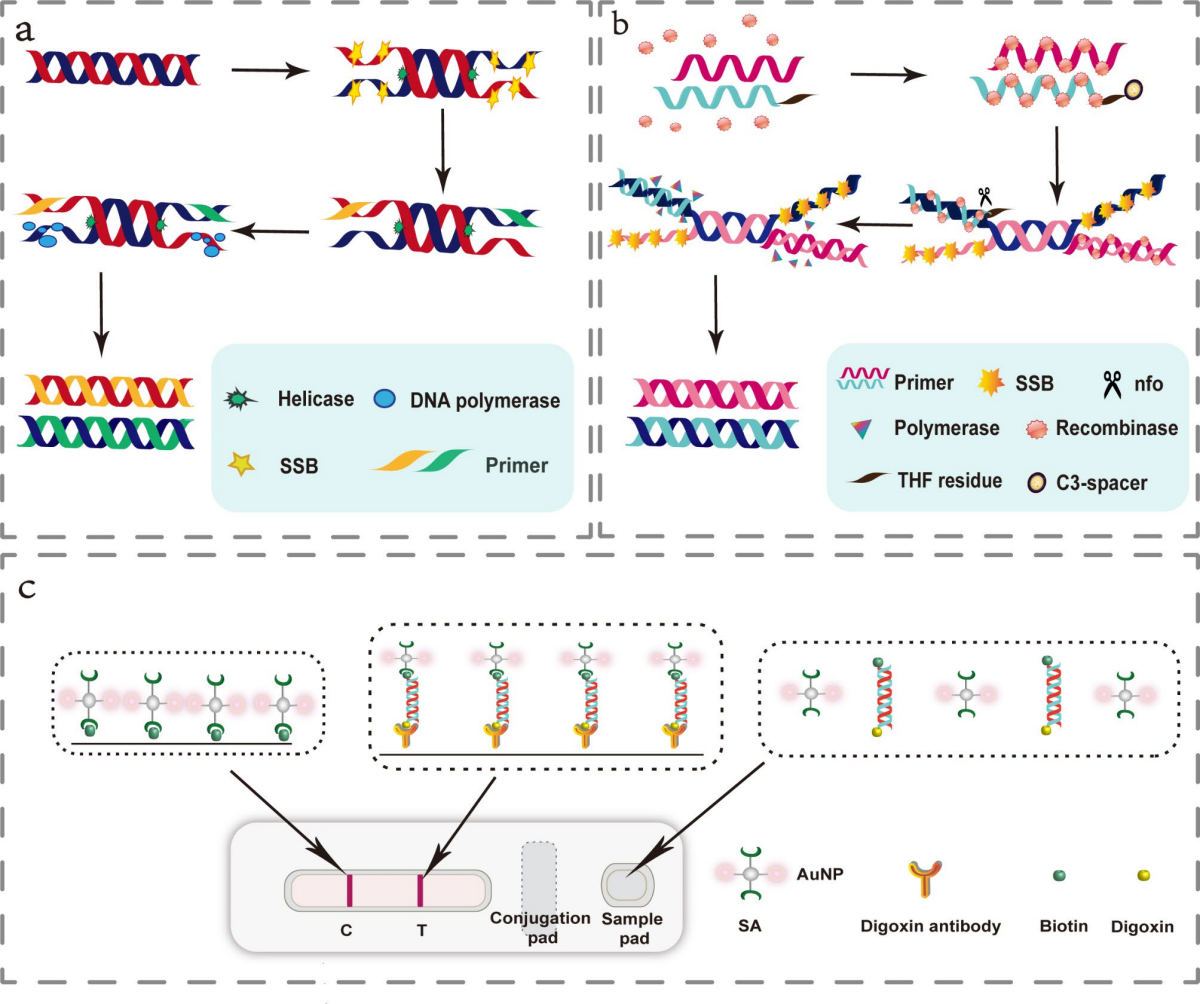
Basic principles of detection technology. Figure 1**a** shows the basic principle of the HDA amplification technique: after the double-stranded DNA is deconvoluted using a helicase, a partial single-stranded sequence is created, and the single-stranded binding protein (SSB) binds to the newly formed single-stranded strand, and subsequent primers bind to the single-stranded strand and amplify it under the action of DNA polymerase. Figure 1**b** shows the basic principle of RPA amplification: Introduce tetrahydrofuran modification sites into the probe and modify the 3 ‘end to prevent elongation. When the probe is paired with the target DNA product amplified by the primer with a marker at the 5 ‘end, Nfo cuts the probe to form free 3’ - OH, which can be used as the primer to continue to extend, and the Synthon generation target DNA sequence; So the target DNA sequence of the offspring will also carry markers. SSB bind to the replaced DNA strand to prevent further replacement. Figure 1**c** shows the basic principle of the LFT technique: the digoxin antibody is fixed to the membrane in a strip (T-line) and the colloidal gold labelling reagent is adsorbed onto the gold pad. When the antigen to be tested is added to the sample pad at one end of the strip, the sample moves forward by capillary action, dissolves the colloidal gold labelling reagent on the binding pad and reacts with each other, and then moves to the area of the fixed antigen or antibody when the conjugate of the antigen to be tested and the gold labelling reagent When the sample is moved to the area of the fixed antigen or antibody, the conjugate of the test article and the gold standard reagent is specifically bound and retained on the test strip, which can be observed by the naked eye

Researchers have developed several isothermal nucleic acid amplification techniques (INAAT) for nucleic acid detection of MPXV [6, 7] in order to address the aforementioned issues and enable MPXV detection to meet the needs of point-of-care testing (POCT). By adding various enzymes and specific primers, INNAT can rapidly amplify the target fragment at a constant temperature without requiring a complex heating procedure. INAAT simplifies the instrument requirements, reduces the reaction time, and satisfies the clinical staff’s demand for quick and convenient nucleic acid detection. Invented in 2004, helicase dependent amplification (HDA) is an in vitro thermostatic gene amplification technique that mimics the replication mechanism of DNA in animals [8]. Figure 1a demonstrates the aforementioned principle. During the reaction, the following three proteins, helicase, single-strand binding protein (SSB), and DNA polymerase, are required. HDA is similar to PCR in that it involves double-stranded DNA unwinding annealing, and extension, with the major difference being the use of enzymes to maintain the single-stranded action as opposed to heating the double-stranded process. In 2006, Piepenburg developed Recombinase polymerase amplification (RPA), a new full-process isothermal amplification technology that uses enzymes to open double-stranded DNA similar to HDA [9]. The principle is demonstrated in Fig. 1b. As with HDA, the same method used to detect PCR products can also be used to detect RPA. RPA possesses the advantages of HDA. Moreover, in comparison to the HDA reaction temperature of 65 ℃, the optimal RPA reaction temperature can be performed at room temperature and maintained between 30 ℃ and 40 ℃ without denaturation, thereby significantly accelerating the rate of reaction. When compared to PCR experiments, not only can both these methods be used in whole process experiments at a constant temperature, but they also have simple operation and fewer instrument and personnel requirements, making them suitable for rapid diagnosis at the grassroots or on-site level. Thus, since their development, HDA and RPA have been used extensively in medical diagnosis, agriculture, food, and biological safety, amongst other fields [10–13]. Moreover, by combining lateral flow test (LFT) and INAAT, the detection results can be observed with the naked eye. The reaction principle is illustrated in Fig. 1c. Furthermore, this method is less expensive than real-time quantitative detection and is ideally suited for detection at the grassroots level.

Herein, three methods for the rapid identification of MPXV were developed and validated: HDA-LFT, RPA-LFT, and real-time quantitative PCR (qPCR). We determined the sensitivity and specificity of the aforementioned three methods and then evaluated their efficacy simulated clinical samples in an effort to develop an efficient test for the early screening and diagnosis of MPXV.

## Materials and methods

### Major reagent materials and equipment

An IsoAmp® II Universal tHDA Kit (NEB, USA) was used for the HDA reaction, while a TwistAmp Basic kit (TwistDX, Cambridge, UK) was utilized to carry out the RPA reaction. The kit used for qPCR was purchased from BioGerm Medical Technology Co. (Shanghai, China). In addition, the following instruments were used: Quantitative real-time PCR system (Thermo Fisher Scientific ABI7500), Electrophoresis system (Bio-Rad Mini-PROTEAN Tetra165-8001), Gel imaging and analysis system (Tanon 5200). The disposable colloidal gold test strips were synthesized by Innovation Biotechnology Co. (Jiangsu, China). Agarose powder was purchased from WISSEN (Beijing, China); SYBR Green I (10,000X) was purchased from Solarbio (Beijing, China), while Endonuclease IV (Nfo) was purchased from Thermo Fisher Scientific. Homologous pseudovirus of monkeypox virus cowpox pseudovirus (ATCCVR-1354D), herpes simplex virus (HSA-BW-039/040/041), variella pseudovirus (VIP (BS) IQC-321), cytogalovirus (HSA-IQC-025/026/027) were purchased from ATCC’s Chinese agent BAIAO INNOVATION. In addition to the above reagents, the MPXV pseudovirus (J691001-0004), nucleic acid extraction kit (B518267-0100), and other reagents were all purchased from Sangon Biotech (Shanghai, China).

### Standard plasmid construction

**Fig. 2:**
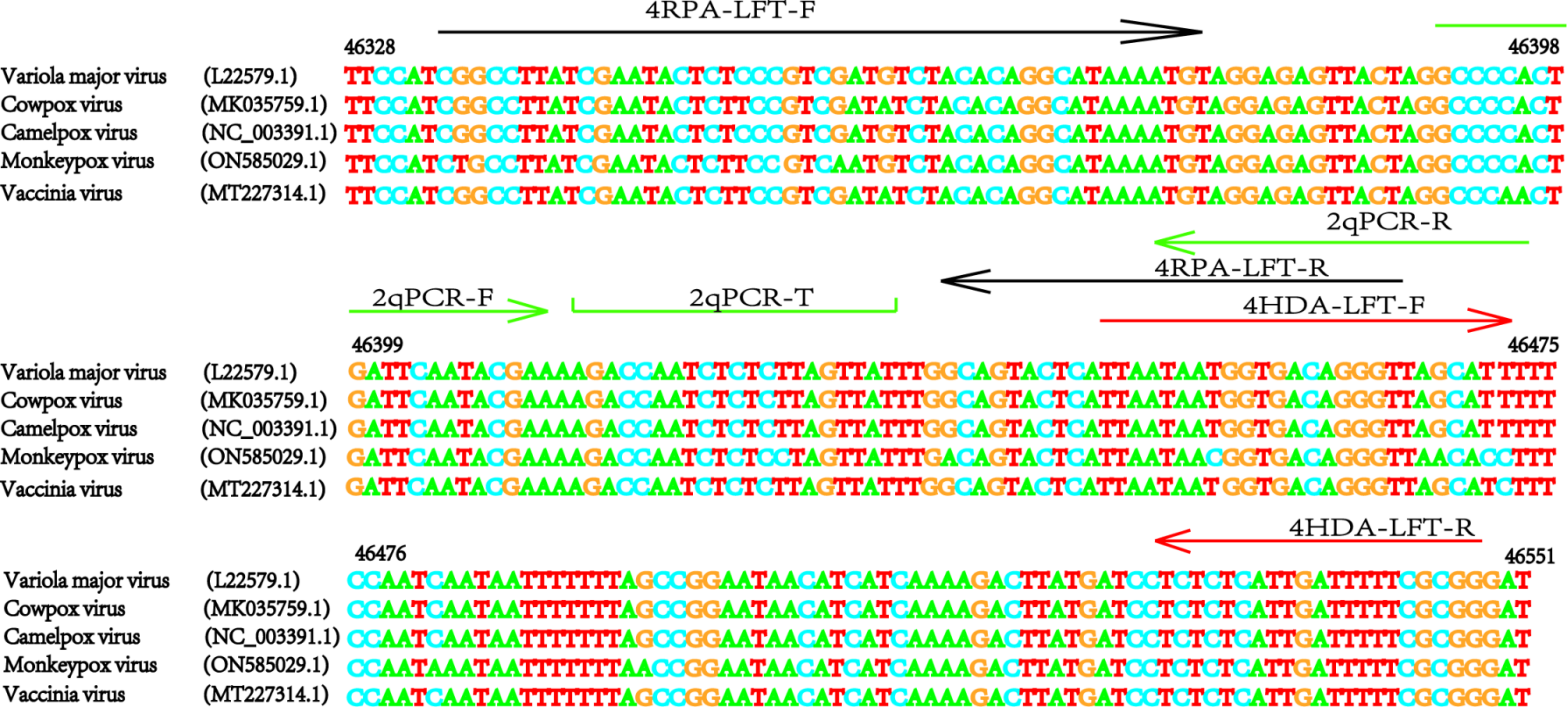
Sequence alignment of MPXV and orthopoxvirus DNA. Compare F3L with DNA target sequences from several orthopox viruses. Virus strains: Monkeypox virus(ON585029.1) consulted on NCBI with Cowpox virus(MK035759.1), Camelpox virus(NC_003391.1), Vaccina virus(MT227314.1) and Variola major virus(L22579.1). F represents forward primer, R represents reverse primer, and T represents probe. Sequences were compared using Jalview and then drawn with Adobe illustrator

We downloaded the entire genome sequence of Monkeypox Virus from NCBI and performed multiple sequence alignments with the Cowpox virus, Camelpox virus, Vaccinia virus, and Variola major virus of the same genus to identify its conserved specific sequence F3L (Monkeypox Virus - F3L Gene ID. ON585029.1) as the subsequent amplification target, ensuring that the designed primers amplified this target site exclusively. This is illustrated in Fig. 2. A synthetic fragment of the monkeypox virus F3L gene (45,667 − 46,128 bp) was inserted into plasmid pUC57 (2710 bp) as a positive template for virus detection. This step was synthesized by GENEWIZ, Inc. (Suzhou, China), and recombinant plasmids were validated by sequencing. Plasmid concentration was measured utilizing a Nanodrop 1000 spectrophotometer (Thermo Fisher Scientific), and the copy size was calculated utilizing the following formula: (6.02 × 10^23^) × (ng/µL × 10^− 9^) / (DNA length × 660) = copies/µL. The plasmids were stored at -80 °C until use. We used the synthesized recombinant plasmid mentioned above to add it to normal human blood samples to optimize the reaction conditions of HDA/RPA-LFT and qPCR, and tested their sensitivity. We extracted the nucleic acid of monkeypox pseudovirus and its homologous pseudoviruses and added them to normal human blood samples to detect their specificity.

### Primers and probes design

According to the primer and probe design scheme introduced in the kit manual, multiple candidate primers were designed using Primer Express 3.0, and the hairpin structures of all primers were evaluated using Origin Evaluator software. The designed candidate primer sequences can be found in the appendix. We selected the pair of primers with the best performance among the candidate RPA primers. In order to improve the specificity and sensitivity of its primers, we added a tetrahydrofuran modified site (THF) as a probe to the forward primer of the basic primer RPA-LFT, and added a C3 spacer at the 3 ‘end to prevent oligonucleotides from being used as amplification primers. When the probe is paired with a target DNA product amplified by a primer with a marker at the 5’ end, Nfo cleaves the probe, forming a free 3’-OH that can be used as a primer to continue the extension and synthesise a daughter target DNA sequence. The corresponding primer and probe markers are shown in Table 1. The primer and probe modifications were synthesized by Biotech Biotechnology (Shanghai, China).

**Table 1:** Sequences of primers and probe for HDA/RPA-LFT and qPCR assay

Primer/Probe^a^	Sequence(5′-3′)	Fluorophore/quencher	Fragment length
4HDA-LFT-F	TTAATAACGGTGACAGGGTTAACACC	5’-Dig 5’-Biotin	102 bp
4HDA-LFT-R	CCGCGAAAAATCAATGAGAGAG		
4RPA-LFT-F	CTGCCTTATCGAATACTCTTCCGTCAATGTCTA/	5’-Dig 5’-Biotin	138 bp
	internal spacer/ACAGGCATAAAATGT-carbon spacer^b^		
4RPA-LFT-R	ACCCTGTCACCGTTATTAATGAGTACTGTC		
2qPCR-F	GCCCCACTGATTCAATACGAA		83 bp
2qPCR-R	AGGTGTTAACCCTGTCACCGTTA		
2qPCR-P	AGACCAATCTCTCCTAGTTAT	5’ 6-FAM/3’-BHQ1	

^a^ HDA-LFT-F, Forward primer; HDA-LFT-R, Reverse primer; qPCR-P, Probe

^b^ internal spacer, A tetrahydrofuran residue; carbon spacer, C3-spacer

### Design of colloidal gold lateral flow strip

In order to observe the products of amplification in a more intuitive manner, we have designed a colloidal gold side flow test strip. The test strip components included a glass cellulose film, a bonding pad, an NC film, an absorbent pad, and a PVC support plate. SA labeled microspheres were sprayed at a rate of 0.8 µL/cm onto the binding pad. Subsequently, digoxin rat monoclonal antibody (1.5 mg/ml) was sprayed on the detection line (T line), and Biotin-BSA was sprayed at a rate of 1 mg/ml on the quality control line (C line). After completing the above steps, they were all dried overnight at 37 ℃. The sample advanced via capillary action, underwent specific binding at the detection site, and was subsequently captured. When the conjugate aggregated on the detection band, the color development outcomes could be observed with the naked eye. Accordingly, a valid test result was indicated by the presence of a red stripe on the C line. On this basis, the T-line red bar indicates positive detection results, whereas the absence of a red bar on the detection line indicates negative detection results.

### Method for HDA/RPA-LFT detection

According to the amplification conditions and reaction system suggested by the kit, the best candidate primers were selected from the designed sets, nucleic acid amplification is performed under the optimized conditions, and sensitivity and specificity were valuated. The final reaction volume of the 50 µL HDA reaction solution contained 5 µL 10X Annealing buffer II, 2 µL MgSO4 (100 mM) and 4 µL NaCl (500 mM), 3.5 µL IsoAmp® dNTP Solution, 3.5 µL IsoAmp® Enzyme Mix, 0.75 µL Forward/Reverse Primer (5 µM), 5 µL of DNA template, and 25.5 µL H_2_O. The final volume of 50 µL RPA reaction solution contained 29.5 µL Primer Free Rehydration buffer, 2.4 µL forward probe (5 µM) and 2.4 µL reverse Primer (5 µM), 8.1 µL H_2_O, 2.5 µL of 280 mM Magnesium Acetate (MgOAc), and 5 µL of DNA template. According to the instructions, the amplification conditions were set to 65 °C for 90 min for HDA and 39 °C for 20 min for RPA. After the amplification reaction was completed, 3 µL was added dropwise to 97 µL PBS buffer for mixing, followed by a drop onto the sample pad of the test strip. The results could be observed between 3 and 5 min later. Each amplification reaction was evaluated in parallel three times, and a blank control was carried out. The above amplified products were combined with the same volume of trichloromethane and centrifuged at 13,000 g for 8 min to determine the accuracy of the LFT results. Approximately 10 µL of the supernatant was combined with 2 µL 6X DNA loading buffer, and 3% agarose gel electrophoresis (AGE) was performed to determine the consistency of the detection results and LFT. We used pure water as a negative control (NC) for the above reactions.

### qPCR assay

A 25 µL qPCR mix system included 16 µL of premix, 0.5 µL of each upstream and downstream primer (10 µM), 1 µL of TaqMan probe (10 µM), 5 µL of template DNA, and 2 µL of water. After adding the aforementioned reaction solutions to the PCR reaction tubes, they were centrifuged and placed in a thermal cycler for amplification. The amplification procedure was as follows: 30 min of denaturation at 94 °C; 5 s of denaturation at 94 °C; 20 s of annealing at 53 °C; and 30 s of extension at 72 °C. The amplification curve was observed after 40 cycles of amplification were performed. The amplified product was electrophoresed on a 1% agarose gel, stained with Ethidium bromide (EB), and subsequently visualized with a gel imaging system. We used pure water as a NC for the above reactions.

## Results

### Selection of primers and optimization of methodology

Among the multiple sets of primers and probes designed for the three aforementioned reactions, the pair of primers with the highest response rate was selected to optimize the reaction conditions. The results of the primer screen are shown in the appendix. The results of AGE after amplification showed that the primers 4HDA-LFT, 4RPA-LFT and 2qPCR amplified well and could be used in the next experiments.

**Fig. 3:**
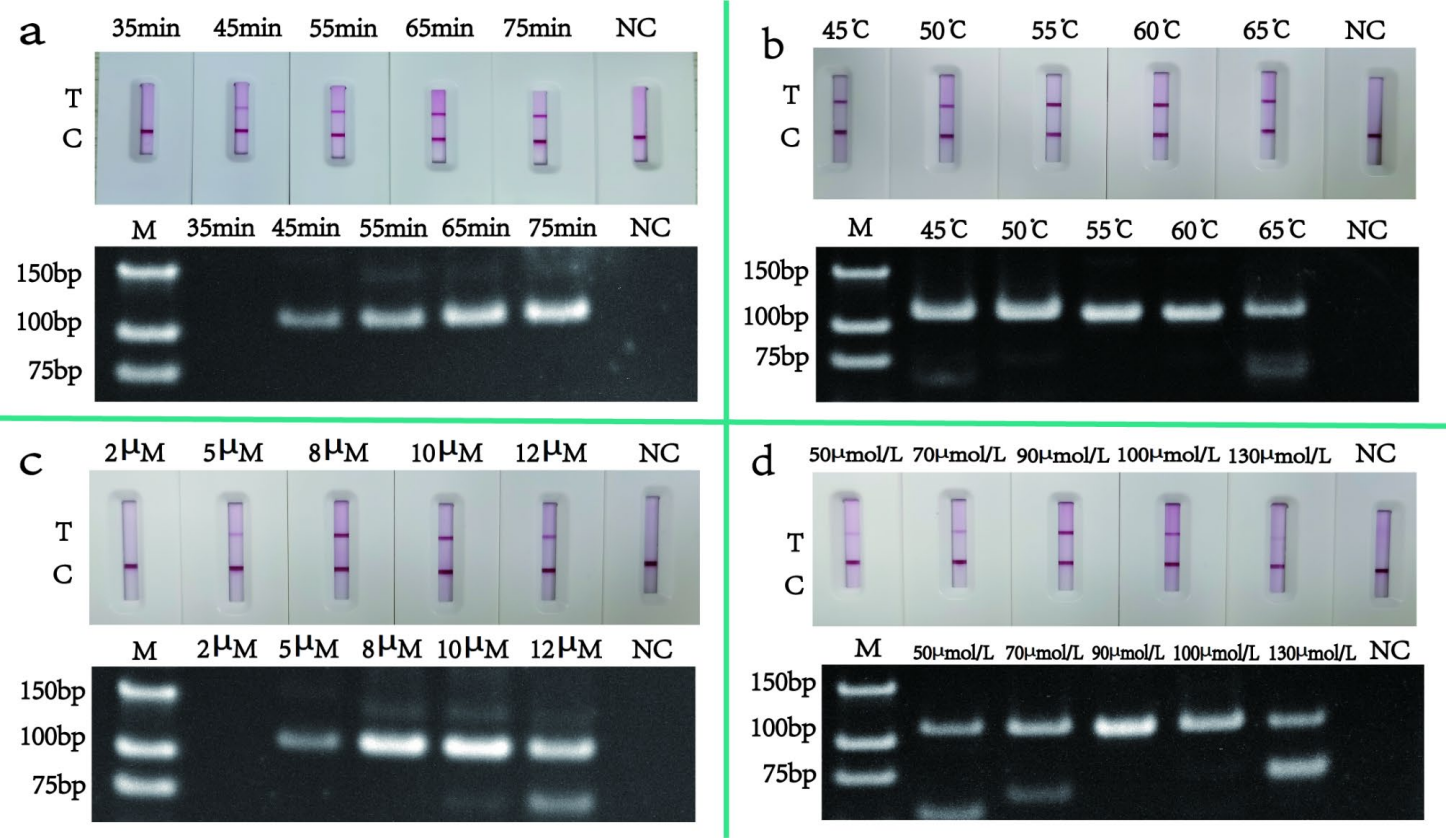
HDA-LFT reaction optimisation. The figure shows the optimisation of the HDA-LFT reaction, where graph figure a show the optimisation of the reaction time, from left to right, with a gradient of 35 − 75 min and the negative control (NC) results respectively. Figure b shows the optimisation of the reaction temperature, from left to right, with a gradient of 45-65 °C and the NC results respectively. Figure c shows the optimisation of primer concentrations, from left to right, with a gradient of 2µM (µmol/L) -12µM and the NC results respectively. Figure d shows the optimisation of dNTPs, from left to right, for 50µmol/L -130µmol/L and the NC results respectively

**Fig. 4:**
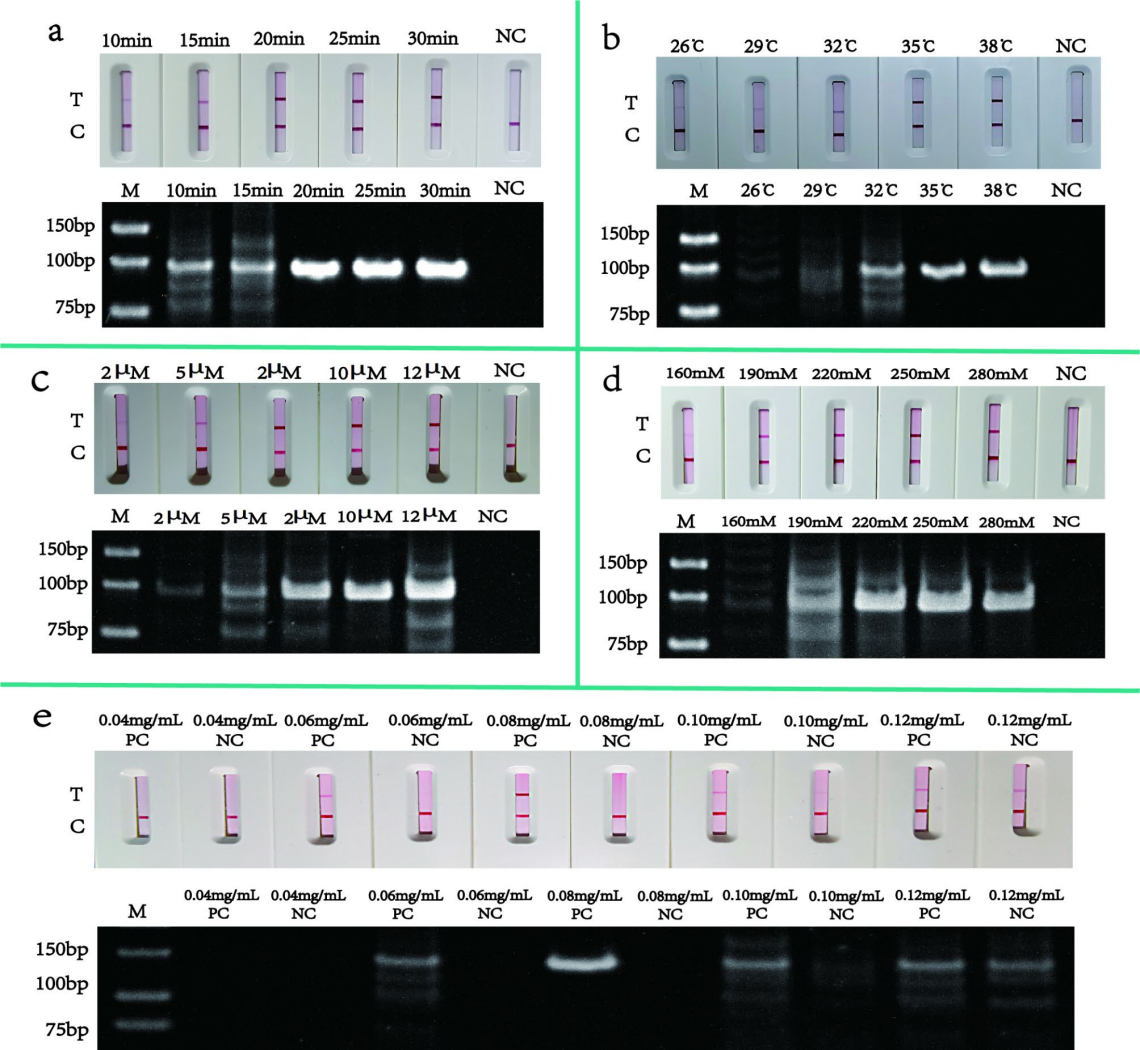
RPA-LFT reaction optimisation. The graph shows the optimisation of the RPA-LFT reaction, where graph a show the optimisation of the reaction time, from left to right, with a gradient of 10 − 30 min, and shows the NC results respectively. Figure b shows the optimisation of the reaction temperature, from left to right, with a gradient of 26-38 °C, and shows the NC results respectively. Figure c shows the optimisation of primer concentrations, from left to right, with a gradient of 2µM -12µM, and NC results respectively. Figure d shows the optimisation of MgOAc, from left to right, for 160 mM -280 mM and NC results respectively. Figure e shows the optimisation of Nfo, from left to right, for 0.04 mg/mL -0.12 mg/mL and NC results, respectively. PC stands for positive control

**Fig. 5:**
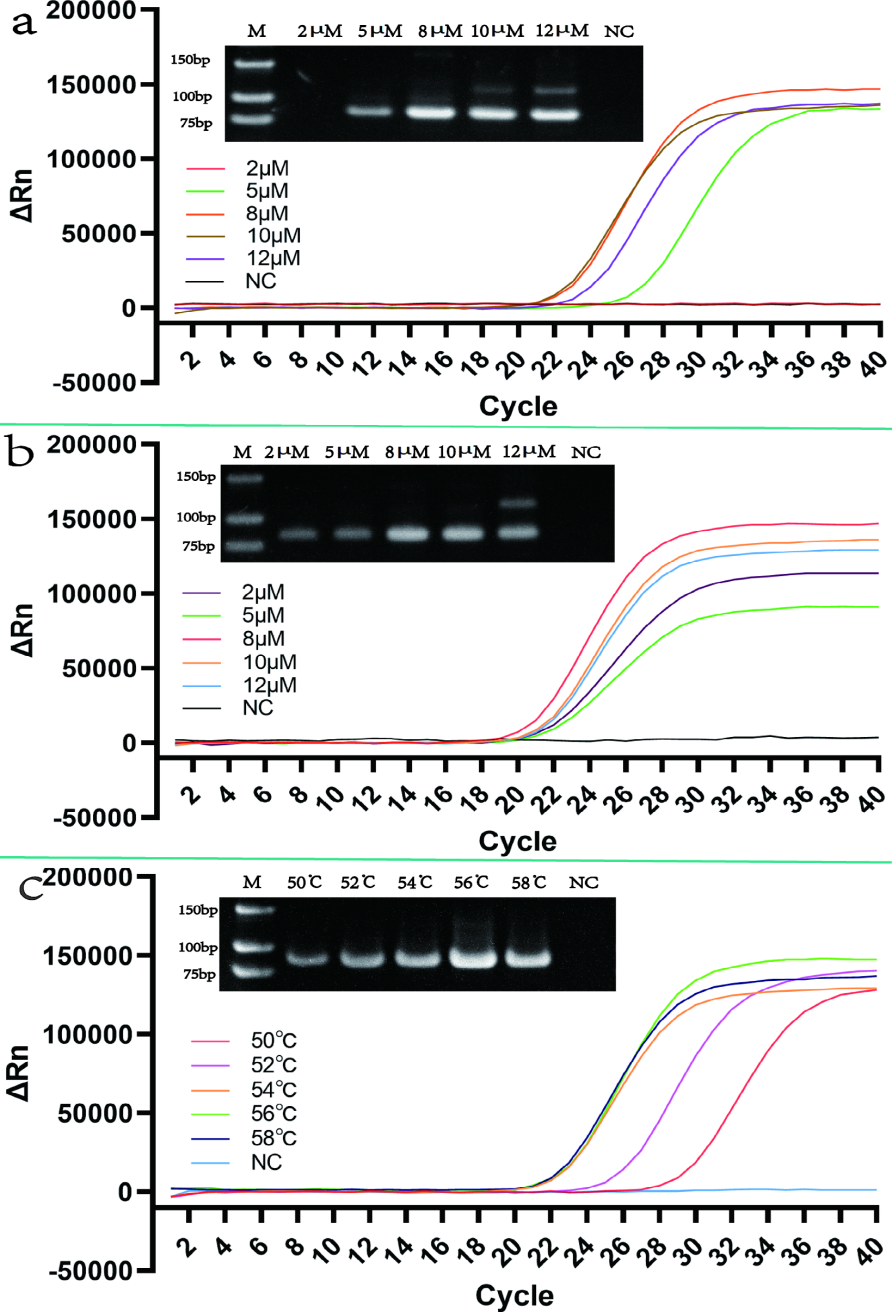
qPCR reaction optimization. The graph shows the optimisation of the qPCR reaction, where graph a show the optimisation of primer concentrations, from left to right, with a gradient of 2µM 12µM and the NC results. Figure b shows the optimisation of probe concentrations, from left to right, with a gradient of 2µM -12µM and the NC results. Figure c shows the optimisation of the annealing temperature, from left to right, with a gradient of 50-58 °C and the NC results

The reaction conditions were optimized for the screened primers. The HDA-LFT reaction was optimized by adjusting the reaction time (35–75 min), reaction temperature (45–65 °C), primer concentration (2 µM–12 µM), and dNTPs concentration (50 µmol/L–130 µmol/L). The results are shown in Fig. 3. Adding 90 µmol/L dNTPs and 8 µM primers under the conditions of 55 °C for 65 min yielded the optimal reaction effect. Subsequently, the reaction time (10–30 min), reaction temperature (26–38 °C), primer concentration (2 µM–12 µM), MgOAc (160 mM–280 mM) and Nfo concentration (0.04 mg/mL–0.12 mg/mL) were optimized in the RPA-LFT reaction. The results of the reactions are shown in Fig. 4. The best results were obtained when 10 µM per primer, 0.08 mg/mL Nfo and 250 mM MgOAc were added to the reaction system and the reaction was carried out at 35 °C for 20 min. In addition, the primer concentration (2 µM–12 µM), the probe concentration (2 µM–12 µM), and the annealing temperature (50 ℃–58 ℃) were optimized in the qPCR reaction. The optimization results are depicted in Fig. 5, and the results obtained after adding 8 µM primers and 8 µM probes to a reaction at 56 ℃ are relatively optimal.

### Sensitivity of HDA/RPA-LFT and qPCR assay

**Fig. 6:**
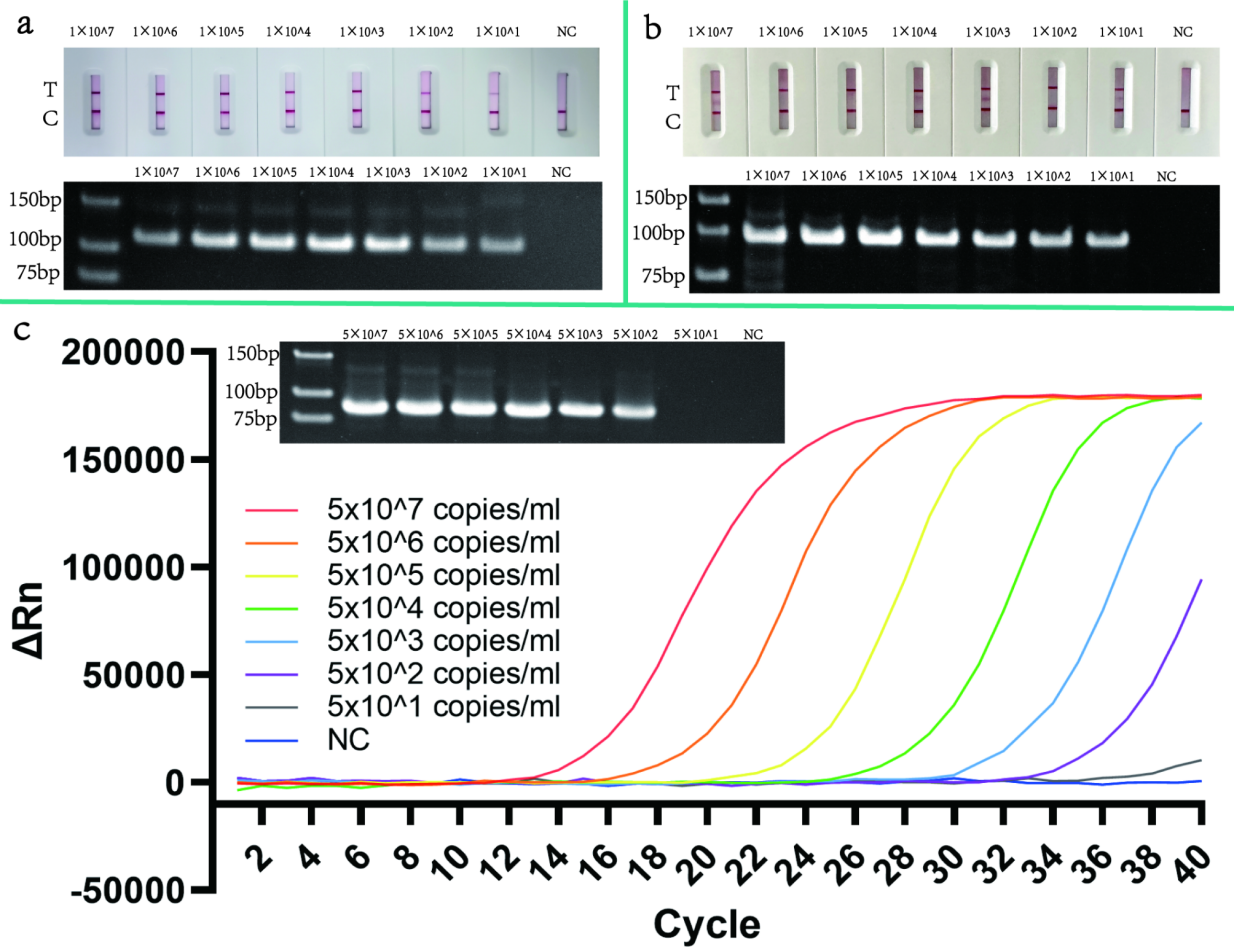
Sensitivity testing of HDA-LFT, RPA-LFT, qPCR. The graph shows the sensitivity detection results for the three methods mentioned above. Figure a shows the sensitivity of the HDA-LFT assay in gradients of 1.0 × 10^7^ copy/µL to 1.0 × 10^1^ copy/µL as well as the NC. Figure b shows the sensitivity of the RPA-LFT assay in gradients of 1.0 × 10^7^ copy/µL to 1.0 × 10^1^ copy/µL and a NC. Figure c shows the sensitivity of the qPCR assay in gradients of 5.0 × 10^7^ copy/mL to 5.0 × 10^1^ copy/mL and NC

**Fig. 7:**
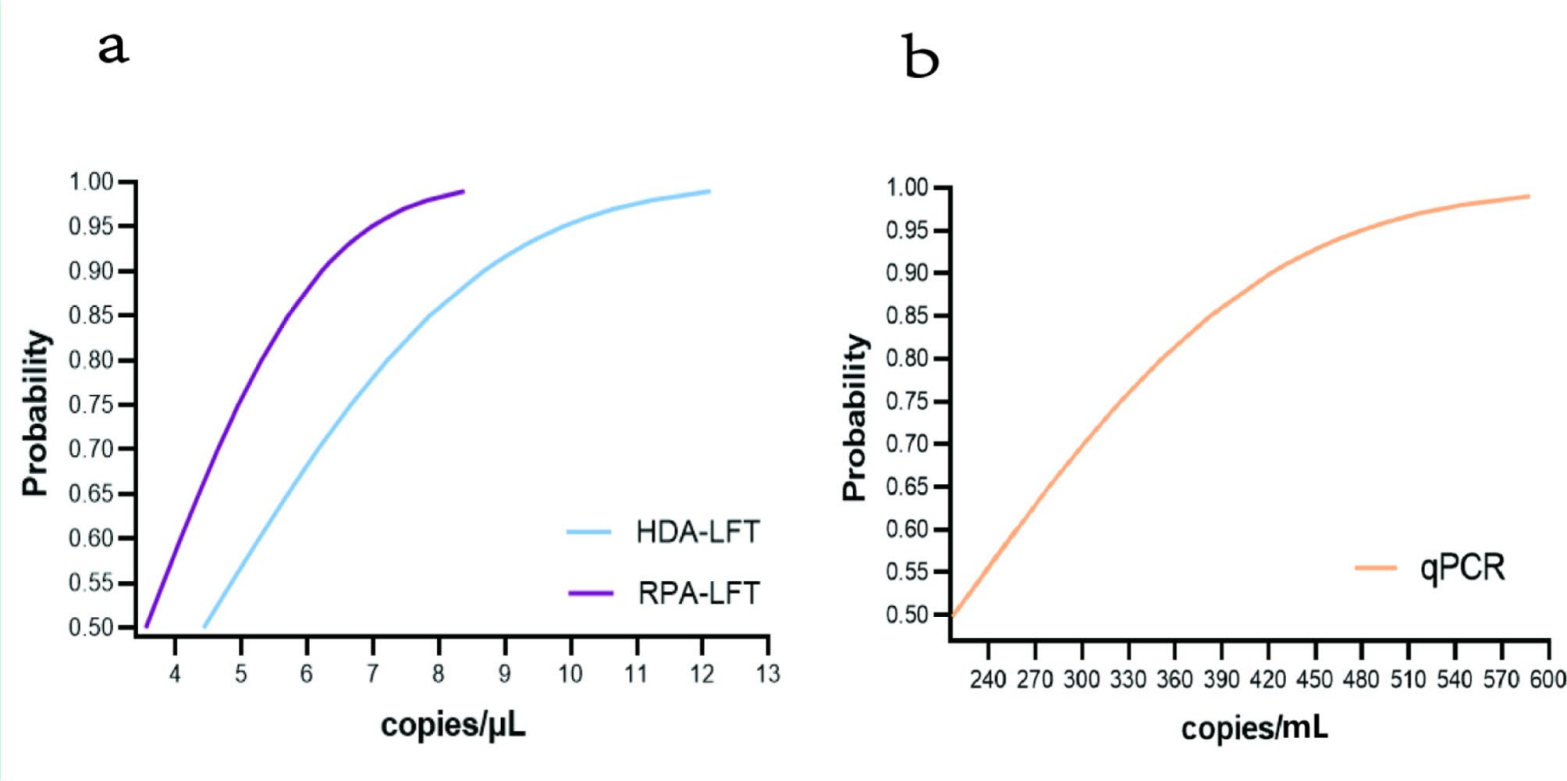
Probit regression analysis for sensitivity analysis of the MPXV assay. Sensitivity analysis was conducted on the three nucleic acid detection methods mentioned above by using recombinant plasmids of different concentrations. The test results were analyzed using SPSS to obtain the above result graph. **A** is the probit analysis result of HDA-LFT and RPA-LFT technology detection sensitivity. **B** is the probit analysis result of qPCR technology detection sensitivity

As reaction templates for assessing the sensitivity of the aforementioned three methods for the detection of monkeypox virus, standard recombinant plasmids were diluted in a gradient from 10^7^ copy/µL to 10^1^ copy/µL. Sensitivity analyses were performed to show that the limit of detection (LOD) of both HDA-LFT and RPA-LFT was less than 10 copies/µL, and the lowest detection limit of qPCR was less than 500 copies/mL. Primer dimers may be generated during the amplification process, which can lead to false positive results and interfere with the evaluation of the assay results. Therefore, the amplification products of the assay were analysed by AGE. The detection results were consistent with those of the AGE method, so the effect of primer dimer could be ignored. The detection results are shown in Fig. 6. To ensure the accuracy of the sensitivity assay, we tested the sensitivity of the three methods using recombinant plasmids with different concentration gradients (1000 copies/µL, 100 copies/µL, 10 copies/µL, 1 copies/µL, 0 copies/µL). 20 parallel experiments were conducted for each concentration using each detection method (Table 2), and the sensitivity of each detection method was evaluated using probit analysis. The detection results are shown in Fig. 7. The sensitivity measurement results indicate that the LOD of the HDA-LFT detection target is 9.86 copies/µL (95% confidence interval, CI 7.52 copies/µL lower bound), the RPA-LFT detection target is 6.97 copies/µL (95% CI 3.90 copies/µL lower bound), and the qPCR detection target is 479.24 copies/mL (95% CI 273.81 copies/mL lower bound).

**Table 2:** Sensitive detection by HDA/RPA-LFT and qPCR assay

Total	HDA-LFT				RPA-LFT				qPCR		
	Positive	Copies (copies/µL)	Positive detection rate		Positive	Copies (copies/µL)	Positive detection rate		Positive	Copies (copies/mL)	Positive detection rate
20	20	1000	100%		20	1000	100%		20	5000	100%
20	20	100	100%		20	100	100%		19	500	95%
20	19	10	95%		20	10	100%		6	50	30%
20	4	1	20%		2	1	10%		1	5	5%
20	1	0	5%		1	0	5%		0	0	0%

Sensitive detection by HDA/RPA-LFT and qPCR assay

### Specificity of HDA/RPA-LFT and qPCR assay

**Fig. 8:**
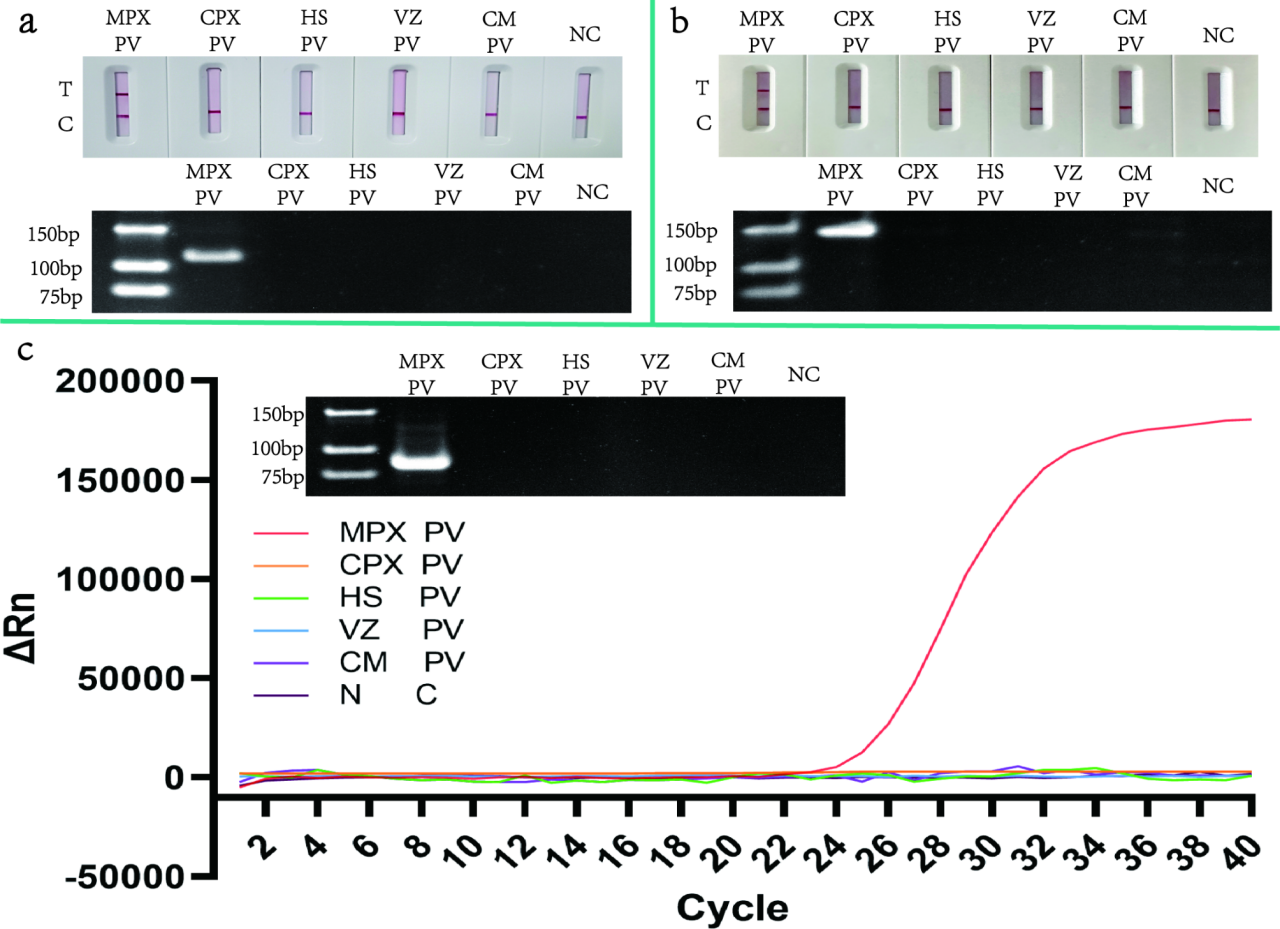
Specificity testing of HDA-LFT, RPA-LFT, qPCR. This figure shows the specificity of the detection results of the three methods mentioned above. The specific detection sequence of the above three methods from left to right is Monkeypox pseudovirus (MPX PV), Cowpox pseudovirus (CPX PV), Herpes-simplex-Virus (HS PV), Varicella pseudovirus (VZ PV), Cytomegalo pseudovirus (CM PV) as well as the NC. Figure a shows the results of HDA-LFT specific detection. Figure b shows the specific results of RPA-LFT, while Figure c shows the specific results of qPCR

Due to the lack of suitable experimental resources, we used a nucleic acid extraction kit to extract DNA templates from monkeypox pseudovirus (MPX PV), cowpox pseudovirus (CPX PV), herpes simplex virus (HS PV), varicella pseudovirus (VZ PV), and cytomegalovirus (CM PV) according to the extraction steps in the manual, and added them to normal blood to simulate real clinical samples to evaluate the specificity of the three detection methods mentioned above. The MPXV pseudovirus concentration used here is 100 copies/µL. In order to analyze its specificity more accurately, we used each detection method to detect a total of 40 samples, including 20 samples containing MPX PV and 20 samples containing other pseudoviruses mentioned above (Table 3). The specificity test results showed that the specificity of HDA-LFT detection target was 94%, RPA-LFT detection target was 90%, and qPCR detection target was 100%. These results indicate that the detection method developed here can detect MPXV with high specificity. The detection results are shown in Fig. 8, the detection results are consistent with the results of AGE.

**Table 3:** HDA/RPA-LFT and q-PCR specific assays

Puedovirus^a^	HDA-LFT		Total		RPA-LFT		Total		qPCR		Total
	Positive	Negative			Positive	Negative			Positive	Negative	
**Positive** ^b^	19	1	20		18	2	20		20	0	20
**Negative** ^c^	3	17	20		1	19	20		2	18	20
**Total**	22	18	40		19	21	40		22	18	40
**Sensitivity**	86%				95%				91%		
**Specificity**	94%				90%				100%		
**Accuracy**	90%				93%				95%		

^a^ Samples containing nucleic acid extracts of MPX PV and other viruses of the same genus

^b^ Simulated clinical samples containing only MPX PV nucleic acid

^c^ Simulated clinical samples without MPX PV nucleic acid (HS PV, VZ PV, CPX PV).

## Discussion

In recent years, MPX has evolved into a global epidemic and a “public health emergency of international concern” [14]. Early in its discovery, MPX was frequently misdiagnosed as smallpox due to the similarity of its symptoms to those of smallpox. Since smallpox has been eradicated, the routine diagnosis of MPX primarily involves distinguishing it from varicella caused by varicella-zoster virus (VZV) [15]. Currently, MPXV is spreading rapidly in non-endemic countries and regions, and confirmed cases of monkeypox have been found successively in Europe and the North. As a crucial step in the process of epidemic prevention and control, the diagnosis and detection of monkeypox require the support of numerous types of diagnosis and detection technologies, rapidity of detection, on-site detection in special locations, clinical diagnosis, laboratory identification, and other work. In addition to developing a gold standard qPCR for the detection of monkeypox virus, we have developed two novel detection methods with high sensitivity and specificity in this study: HDA-LFT and RPA-LFT. The two aforementioned methods are simple to operate; moreover, the instrument is simple to use, test results can be determined by observing the number of lines displayed on the LFT, and the entire testing procedure can be performed without the need for specialized training. Therefore, these two types of methods are anticipated to become ideal diagnostic tools for detecting MPXV in settings with inadequate medical care and limited resources.

Although the definition of a suspected case of MPX and the manner in which MPXV is diagnosed varies slightly across the globe, it can be categorized as follows: (1), Electron microscopy. Th primary advantage of this method is that the results can be observed directly without the use of specific biological reagents; however, the cycle is longer, sample preparation and operation are more complicated, and a laboratory employing an electron microscope must be equipped with qualified technical personnel. (2), molecular diagnosis [16]. These include qPCR and next-generation sequencing (NGS). qPCR is the method of choice for the routine diagnosis of pathogenic microorganisms and the gold standard for the positive diagnosis of MPXV. It has high sensitivity and specificity for detecting MPXV, MPXV branches can be effectively identified, and other OPXV can be distinguished, but the amplification process requires three steps: Denaturation, annealing, and extension, this process requires temperature control equipment, a considerable amount of time, and skilled labor [17]. By detecting the DNA sequence of MPXV, NGS can help us better understand the epidemiology, source of infection, and mode of transmission of the virus. However, NGS is not suitable for large-scale testing because it is expensive and requires the capability to process sequencing data downstream [18]. (3), the detection of serum antibodies, ELISA is the preferred method for serum antibody detection, utilizing antigen-antibody specific binding reaction for immune reaction qualitative and quantitative analysis; thus, the MPXV specific antibody can be detected in either animal or human serum. However, this method is incapable of achieving early diagnosis and can only be used for late auxiliary diagnosis with poor compliance [19]. (4), MPXV’s isolation and culture. Although isolation and culture of MPXV is the gold standard for diagnosing viral disease, the isolation and culture of MPXV is limited and requires a high level of laboratory biosafety; isolation and culture operations must be performed in a Level III or higher laboratory by experienced personnel [20]. Therefore, it is essential to develop a rapid detection method with high sensitivity for MPXV detection in environments with limited resources.

While using the HDA method to detect MPXV for the first time, we also combined it with RPA technology and LFT to determine the sensitivity and specificity of the three detection methods in conjunction with our own qPCR. Other INAAT have been tested for the monkeypox virus before [7, 21]. Loop-mediated isothermal amplification (LAMP) is a novel technique for the amplification of nucleic acids that does not involve thermal cycling. Using LAMP technology, Iizuka et al., established a real-time quantitative amplification system for the monkeypox virus genome. Primers were designed to target the type A inclusion body (AT1), the D14L gene specific to Congo Basin monkeypox virus, and a portion of the AT1 gene from West African MPXV. LAMP was used to distinguish the strain from the Congo Basin from the strain from West Africa [22]. Although LAMP is a convenient, efficient, and inexpensive technique for detection [23, 24], the technique itself requires a significant amount of optimization and validation to identify specific primers and enzymes, as well as the design of many pairs of primers and enzymes. In addition, DNA polymerases are more sensitive to changes in temperature, prone to false positive results, and prone to the formation of difficult to eliminate aerosols [25, 26]. Compared to the LAMP assay, our HDA-LFT and RPA-LFT assays offer significant advantages. Simple primer composition, which eliminates the need for multiple sets of primers, effectively reduces the formation of dimers. LAMP technology’s high temperature amplification increases the likelihood of cross-contamination, whereas the reaction temperature of RPA is closer to that of the human body, can effectively avoid aerosol pollution, and can be used outside of the PCR laboratory. Combining HDA technology and RPA technology with LFT not only has high sensitivity and specificity but also simplifies the operation steps, does not require complex equipment or professional training, and allows for more direct observation of reaction results; therefore, this study has the potential to become an ideal tool for POCT [22].

In this study, the specific sequence F3L of MPXV was detected using HDA-LFT, RPA-LFT, and qPCR techniques. The reaction temperature, reaction time, primer concentration, and dNTPs concentration were optimized for HDA technology. As can be seen from the optimisation results, the concentration of dNTPs has an impact on the outcome of the reaction and when it is high, it inhibits the reaction and leads to the production of primer dimers. Additionally, we have optimized the reaction time, temperature, concentration of primers, MgOAc, and Nfo in the RPA technique. The Nfo enzyme, the probe shear enzyme of the RPA technique, has a significant impact on the efficacy of the reaction, with higher Nfo concentrations resulting in false positive results. Moreover, in designing conventional qPCR reactions, we optimized annealing temperature, primer concentration, and probe concentration. Due to our inability to obtain clinical samples related to MPX for our research, we simulated the method of adding recombinant plasmids to normal blood samples to detect real clinical samples to address this issue. This is due to the fact that the virus can be detected in the blood at an early stage of the infection process. This stage is called the prodromal stage and can occur before visible skin lesions. The results of the sensitivity test showed that the LOD of the HDA-LFT detection target is 9.86 copies/µL (95% CI 7.52 copies/µL lower bound), the RPA-LFT detection target is 6.97 copies/µL (95% CI 3.897 copies/µL lower bound), and the qPCR detection target is 479.24 copies/mL (95% CI 273.81 copies/mL lower bound). We used MPX PV, CPX PV, HS PV, VZ PV and CMPV as targets to verify the specificity of the above three methods. The reaction results showed that the specificity of the above three methods in detecting Monkeypox virus homologues was more than 90%.

Although this study demonstrated many advantages of the HDA-LFT and RPA-LFT techniques in the detection of MPXV, we acknowledge that there are some limitations: firstly, the results of the LFT are usually judged by visual observation of the appearance of bands, which not only results in the assay providing only a qualitative assessment of the response, but also makes it difficult to obtain accurate experimental results due to inter-individual variability in judgement. Secondly, although HDA/RPA-LFT is simpler than the traditional PCR method, and does not need a complex temperature change process, it can be detected in a water bath or at room temperature, but it can only be used after the sample is pretreated with nucleic acid extraction reagents and special Laboratory equipment, which may weaken the applicability in this field. In future research, we will conduct more in-depth research on how to use HDA/RPA-LFT technology to directly detect MPXV clinical samples, hoping to provide effective reference value for promoting the application of MPXV detection technology in POCT in the future. Finally, the potential contamination is a drawback of the above detection methods, which may be caused by opening the reaction tube when analyzing the product through LFT. Therefore, it is crucial to take sealing measures during the analysis process to prevent potential pollution. Besides, a point of concern is that the sensitivity LOD of the qPCR technique in our experiments as a control group showed some differences compared with the sensitivity of the recently reported qPCR assays on MPXV [27], which may be related to the quality of the reagents, the accuracy of the instruments and the technical proficiency of the operators as well as the design of the primers. We will continue to explore and optimise these differential factors in depth in subsequent experiments. In the future biosecurity prevention and control process, it will be necessary to further establish a monkeypox virus detection platform, develop efficient and accurate detection products, continually improve detection efficiency and accuracy, and establish strategic technical reserves to prevent safety accidents. It can prevent the occurrence of major public health incidents.

## Conclusion

In this study, we developed a convenient, highly sensitive and specific HDA-LFT, RPA-LFT, and qPCR assay for the detection of MPXV, which we hope will serve as a solid foundation for future applications in the early screening of MPX, its diagnosis, and evaluation of its viral efficacy.

### Electronic supplementary material

Below is the link to the electronic supplementary material.

Supplementary Material 1

Supplementary Material 2

## Data Availability

The datasets supporting the conclusions of this article (are) included within the article and supplementary materials.
