# Supplementary material for: Detection of monkeypox virus using helicase dependent amplification and recombinase polymerase amplification combined with lateral flow test
Source: Virol J. 2023 Nov 23;20:274. doi: 10.1186/s12985-023-02223-8 (PMC10668421; doi:10.1186/s12985-023-02223-8)
Supplement: Supplementary file 1 — Supplementary Material 1 [file 12985_2023_2223_MOESM1_ESM.docx]

**Monkeypox virus F3L sequence (5′-3′)**

TCAGAATCTAATGATGACATAACTAAGAAGTTTATCTACAGCCAATTTAGCTGCATTATTTTTAGCATCTCGTTTAGATTTTCCATCTGCCTTATCGAATACTCTTCCGTCAATGTCTACACAGGCATAAAATGTAGGAGAGTTACTAGGCCCCACTGATTCAATACGAAAAGACCAATCTCTCCTAGTTATTTGACAGTACTCATTAATAACGGTGACAGGGTTAACACCTTTCCAATAAATAATTTTTTTAACCGGAATAACATCATCAAAAGACTTATGATCCTCTCTCATTGATTTTTCGCGGGATACATCATCTATTATAGCATCAGCATCAGAATCTGTAGGCCGTGTATCAGCATCCATTGTCGTAGACCAACGAGGAGGAGTATCGTTGGAGCTGTAAACCATAGCACTACGTTGAAGATCATACAGAGCTTTATTAACTTCTCGCTTCTCCAT

**HDA-LFT primer design**

Note: After the initial screening of the primer, it was modified.

| **Primer/Probe** | **Sequence(5′-3′)** | **Fragment length** |
| --- | --- | --- |
| 1HDA-LFT-F | CTGATTCAATACGAAAAGACCAATC | 65bp |
| 1HDA-LFT-R | TGTCACCGTTATTAATGAGTACT |  |
| 2HDA-LFT-F | AATAACGGTGACAGGGTTAACACC | 100bp |
| 2HDA-LFT-R | CCGCGAAAAATCAATGAGAGAG |  |
| 3HDA-LFT-F | TCGCGGGATACATCATCTATTATAGC | 87bp |
| 3HDA-LFT-R | TCCTCCTCGTTGGTCTACGACAAT |  |
| 4HDA-LFT-F | TTAATAACGGTGACAGGGTTAACACC | 102bp |
| 4HDA-LFT-R | CCGCGAAAAATCAATGAGAGAG |  |
| 5HDA-LFT-F | CTGCCTTATCGAATACTCTTCCGTCAATGTCTA | 138bp |
| 5HDA-LFT-R | ACCCTGTCACCGTTATTAATGAGTACTGTC |  |

**RPA-LFT primer design**

Note: After the initial screening of the primer, it was modified.

| **Primer/Probe** | **Sequence(5′-3′)** | **Fragment length** |
| --- | --- | --- |
| 1RPA-LFT-F | TTTGACAGTACTCATTAATAACGGTGACAGGG | 141bp |
| 1RPA-LFT-R | CTGATGCTATAATAGATGATGTATCCCGCGAA |  |
| 2RPA-LFT-F | CCTTATCGAATACTCTTCCGTCAATGTCTAC | 139bp |
| 2RPA-LFT-R | GTTAACCCTGTCACCGTTATTAATGAGTACTGTC |  |
| 3RPA-LFT-F | TATCGAATACTCTTCCGTCAATGTCTACACAGG | 133bp |
| 3RPA-LFT-R | AACCCTGTCACCGTTATTAATGAGTACTGTC |  |
| 4RPA-LFT-F | CTGCCTTATCGAATACTCTTCCGTCAATGTCTA | 138bp |
| 4RPA-LFT-R | ACCCTGTCACCGTTATTAATGAGTACTGTC |  |
| 5RPA-LFT-F | TTGACAGTACTCATTAATAACGGTGACAGGG | 138bp |
| 5RPA-LFT-R | GATGCTATAATAGATGATGTATCCCGCGAA |  |

**q-PCR primer and probe design**

Note: After the initial screening of the primer, it was modified.

| **Primer/Probe** | **Sequence(5′-3′)** | **Fragment length** |
| --- | --- | --- |
| 1qPCR-F | ATACTCTTCCGTCAATGTCTACAC | 97bp |
| 1qPCR-R | CAAATAACTAGGAGAGATTGG |  |
| 1qPCR-T | GCATAAAATGTAGGAG |  |
| 2qPCR-F | GCCCCACTGATTCAATACGAA | 83bp |
| 2qPCR-R | AGGTGTTAACCCTGTCACCGTTA |  |
| 2qPCR-T | AGACCAATCTCTCCTAGTTAT |  |
| 3qPCR-F | TTGTCGTAGACCAACGAGGAGG | 96bp |
| 3qPCR-R | TGGAGAAGCGAGAAGTTAATAAAGC |  |
| 3qPCR-T | GTATCGTTGGAGCTGTAAACC |  |
| 4qPCR-F | TCCGTCAATGTCTACACAGGC | 127bp |
| 4qPCR-R | AGGTGTTAACCCTGTCACCG |  |
| 4qPCR-T | TAAAATGTAGGAGAGTTACTA |  |
| 5qPCR-F | ATCTGCCTTATCGAATACTCTTC | 108bp |
| 5qPCR-R | ATAACTAGGAGAGATTGGTCTT |  |
| 5qPCR-T | GTCAATGTCTACACAGG |  |

**Primer screening map for HDA-LFT:**


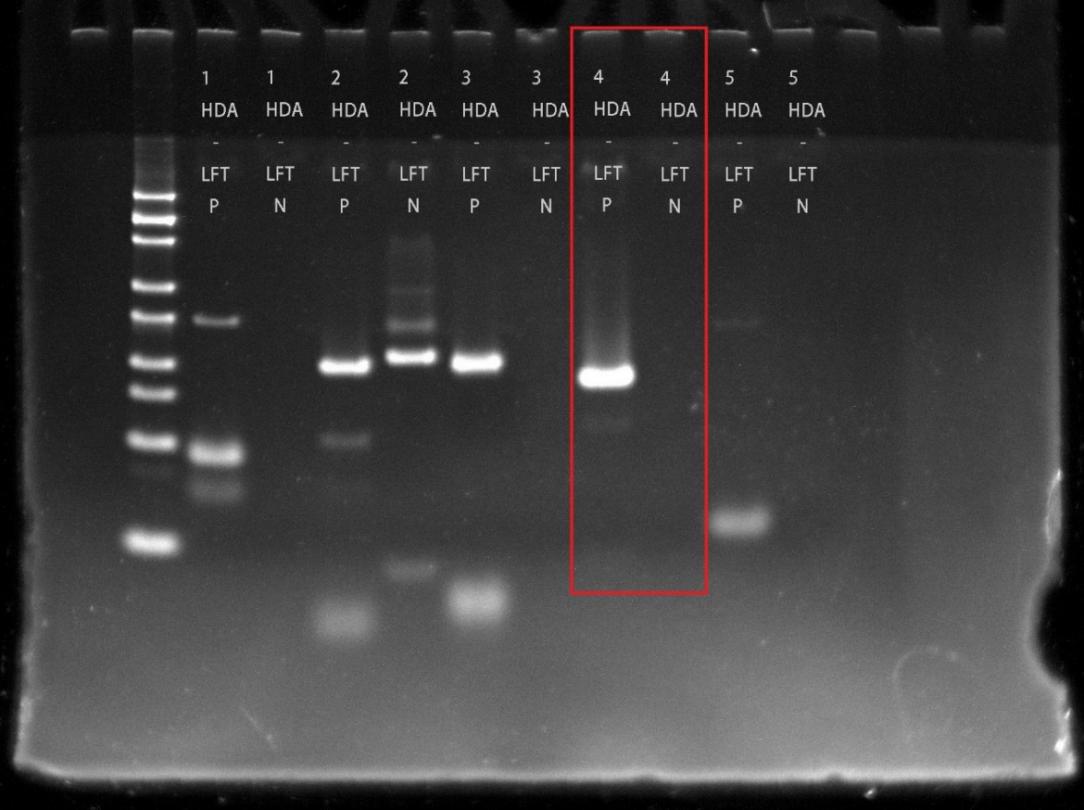


**Primer screening map for RPA-LFT:**


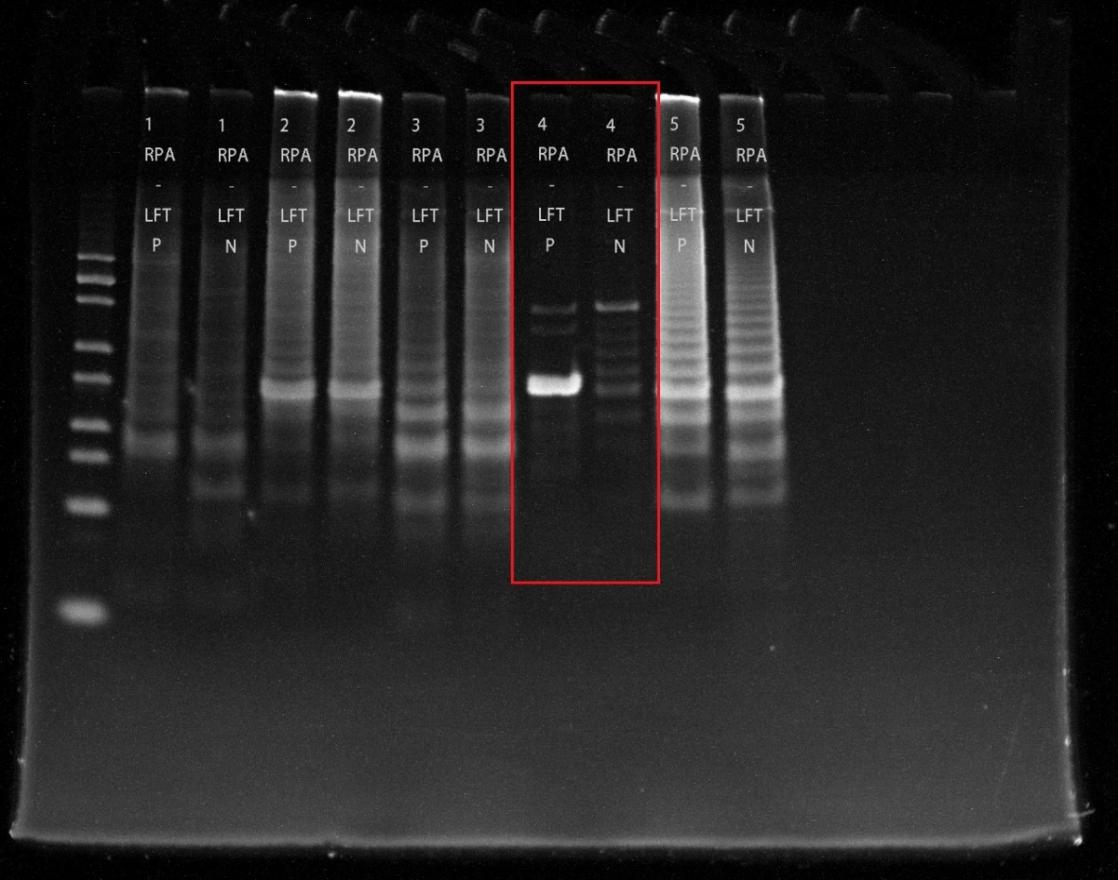


**Primer screening map for qPCR:**


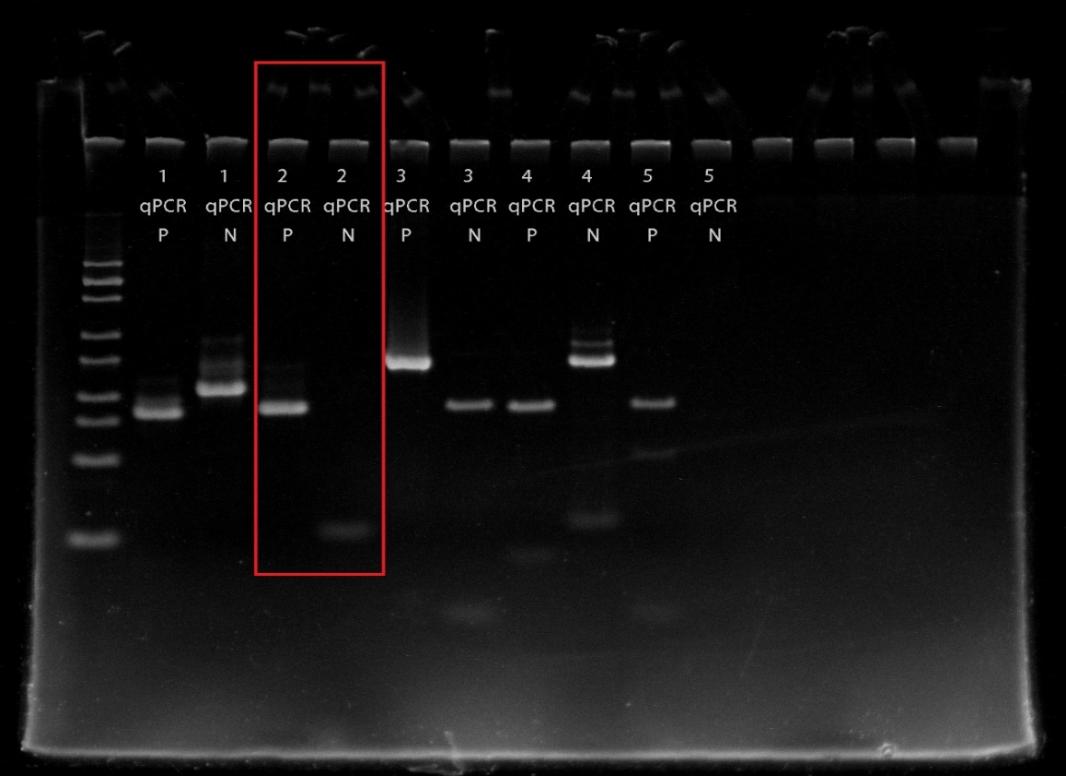


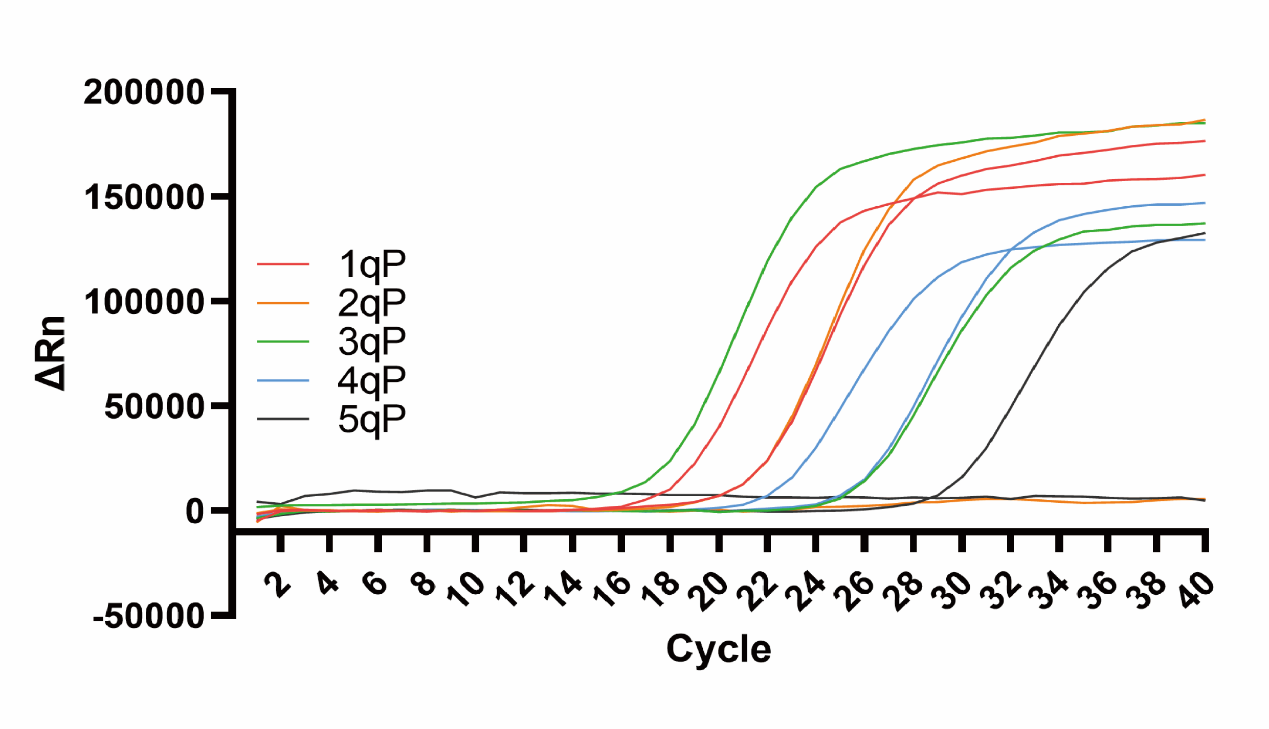


**HDA reaction time optimisation results**

**
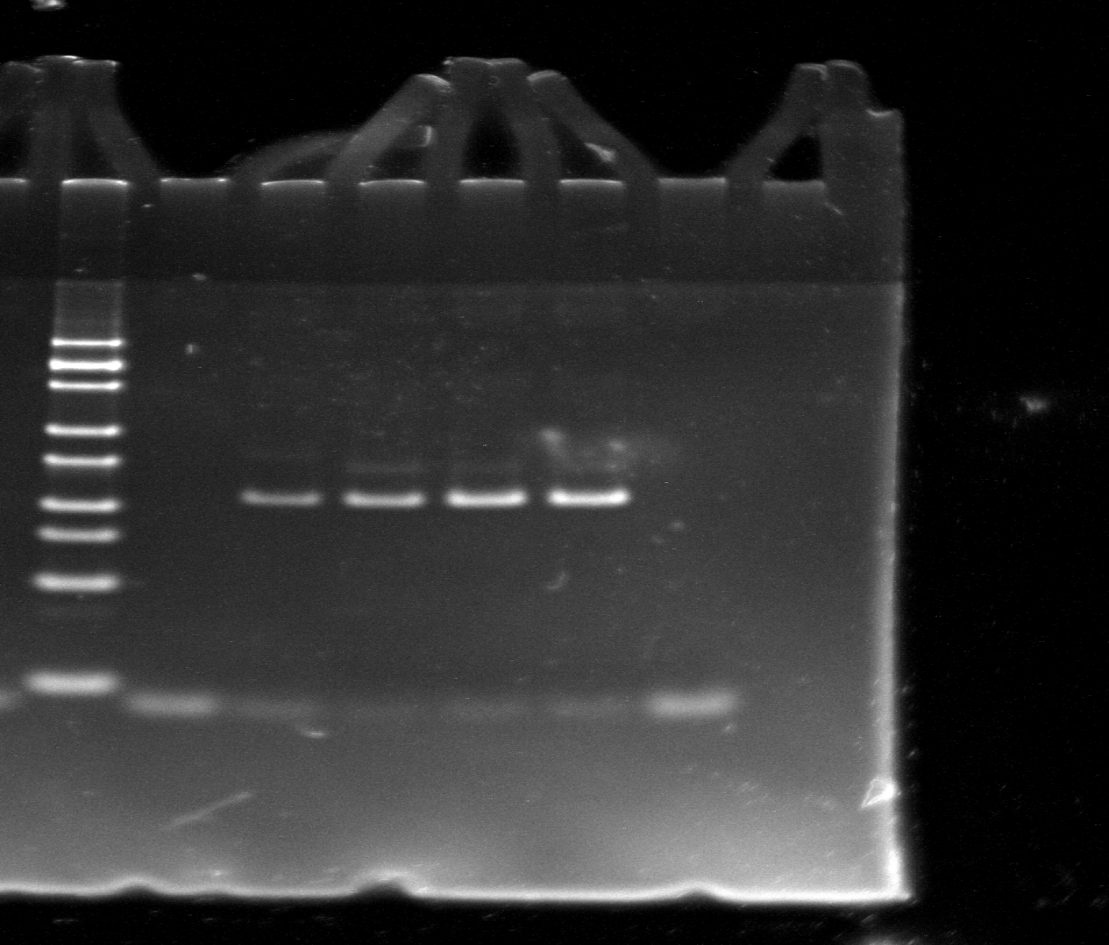
**

**HDA reaction temperature optimisation results**

**
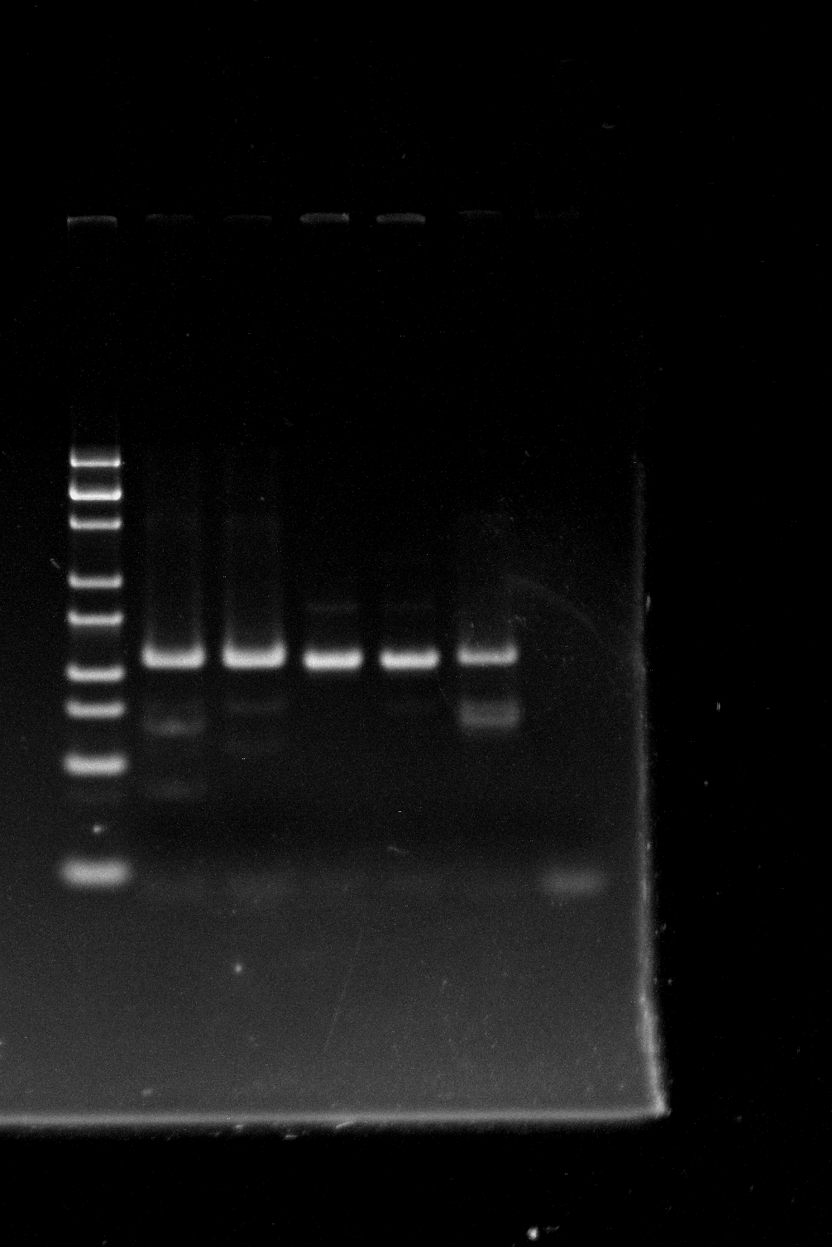
**

**HDA primer concentration optimization results**

**
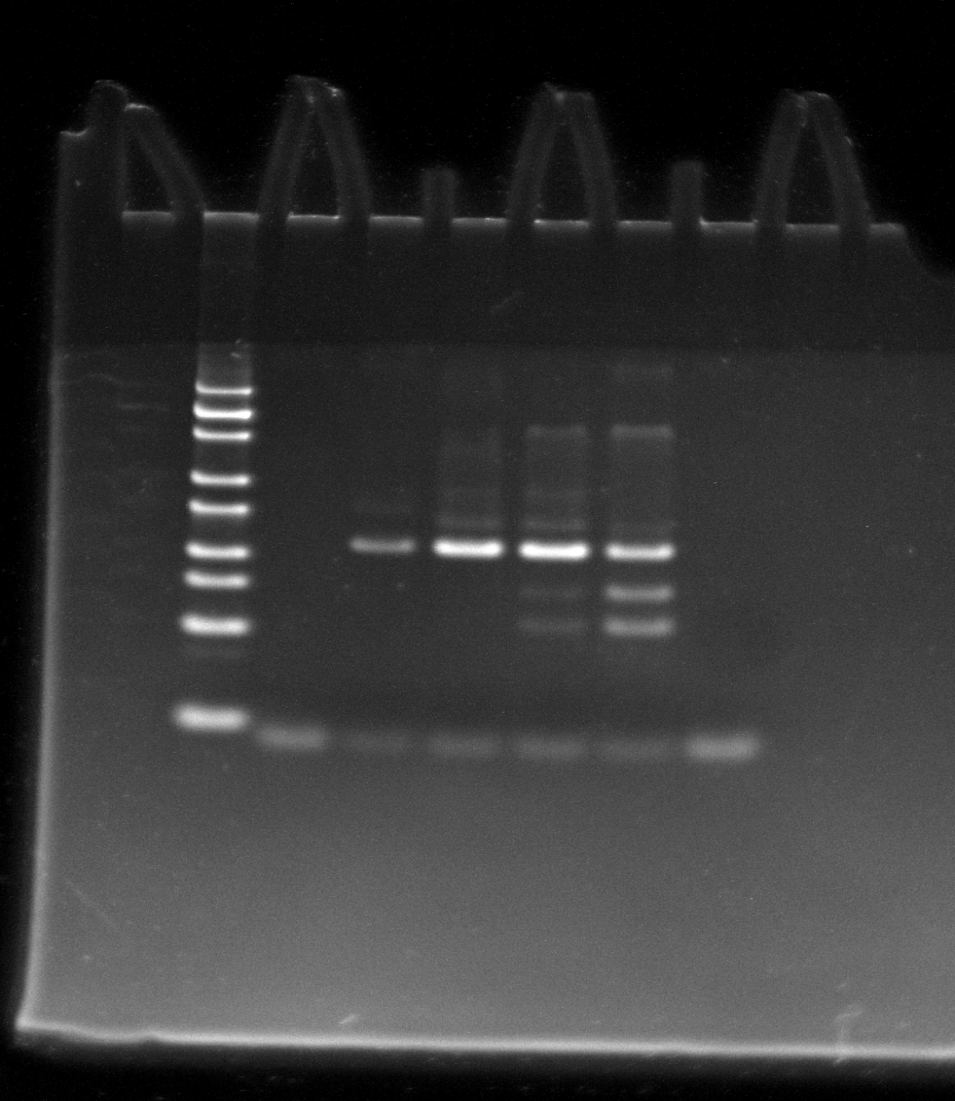
**

**HDA dNTPs concentration optimization results**

**
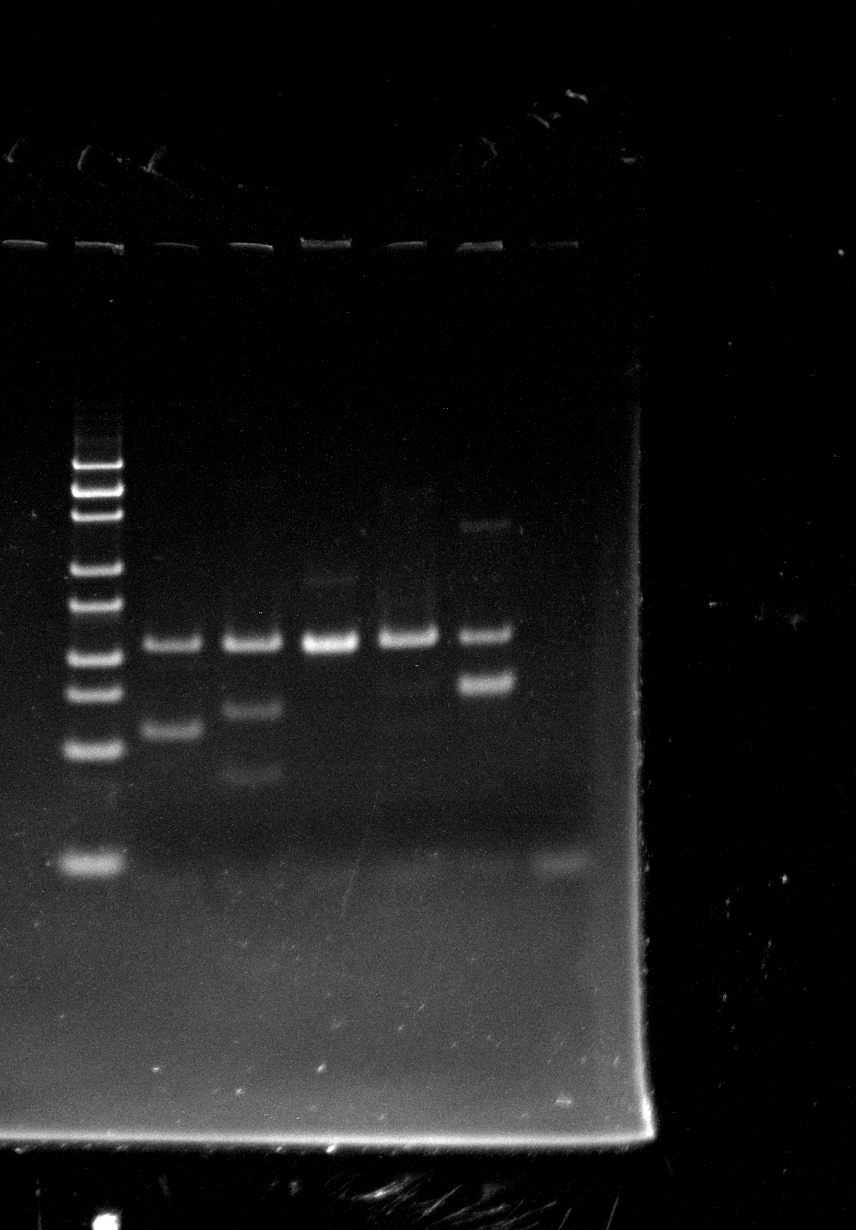
**

**RPA reaction time optimisation results**

**
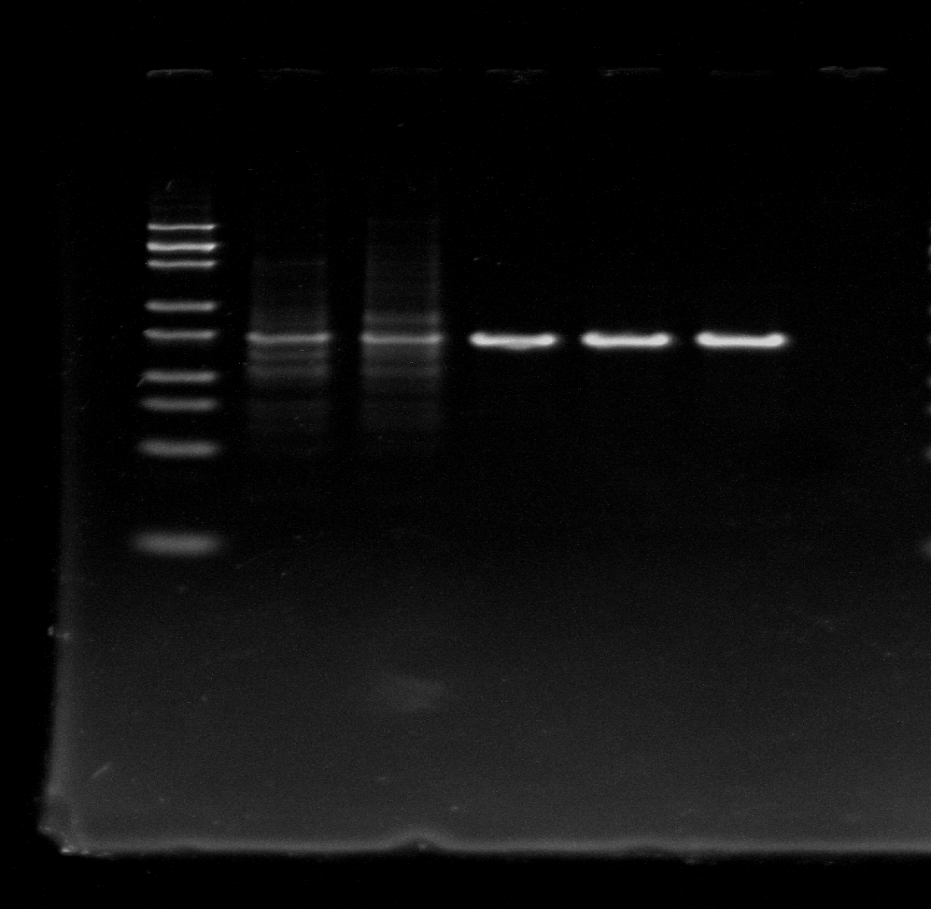
**

**RPA reaction temperature optimisation results**

**
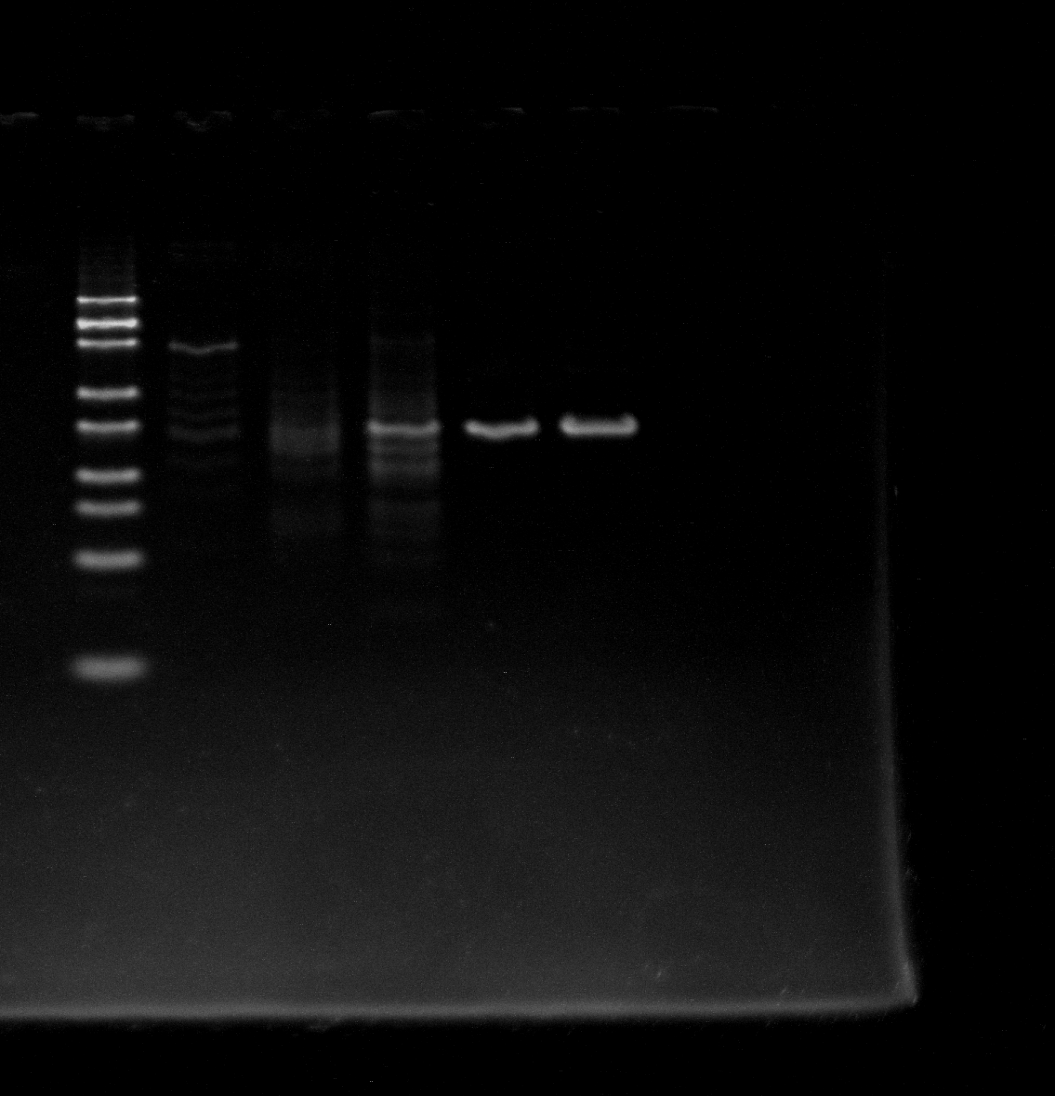
**

**RPA primer concentration optimization results**

**
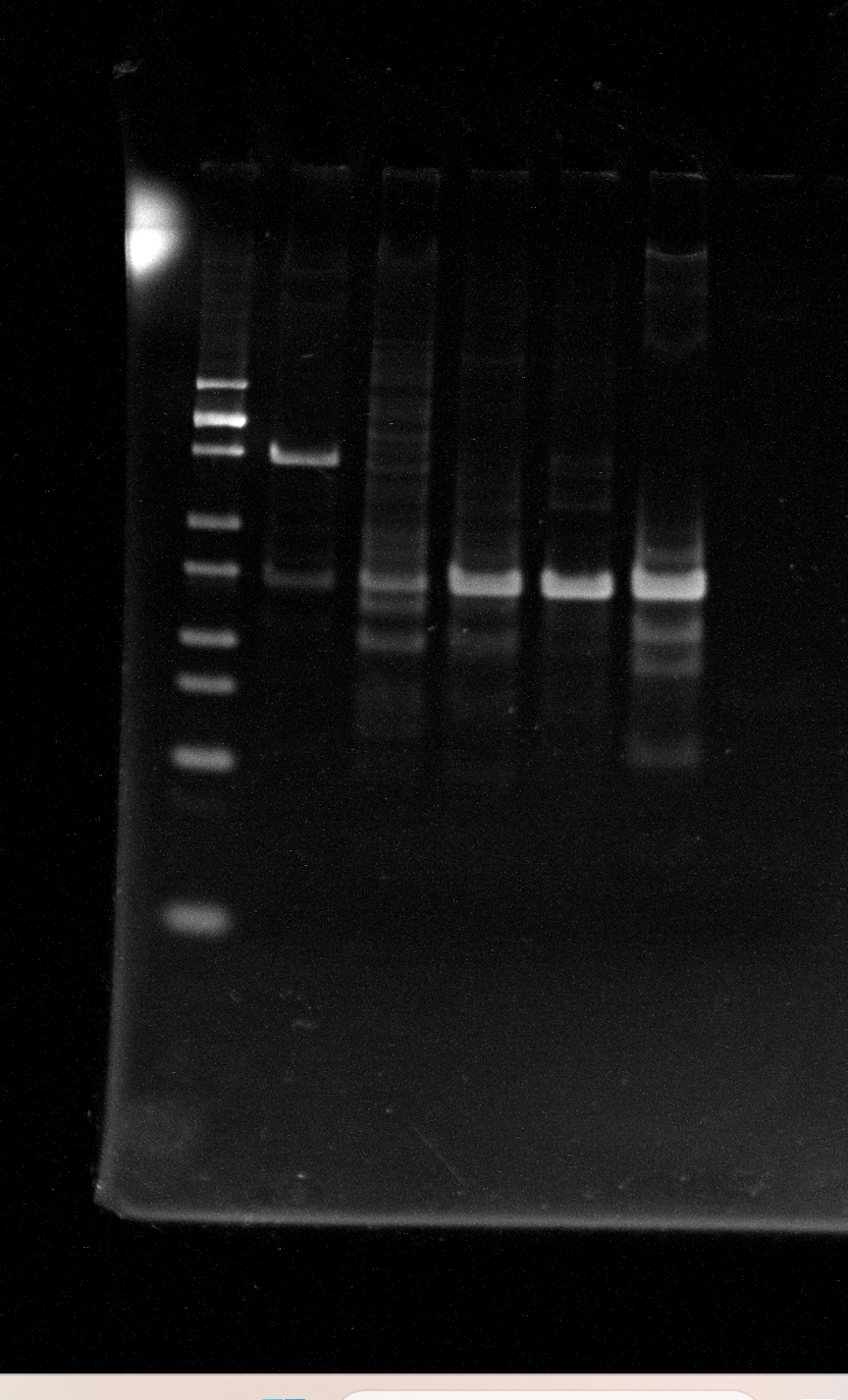
**

**RPA MgOAc concentration optimization results**

**
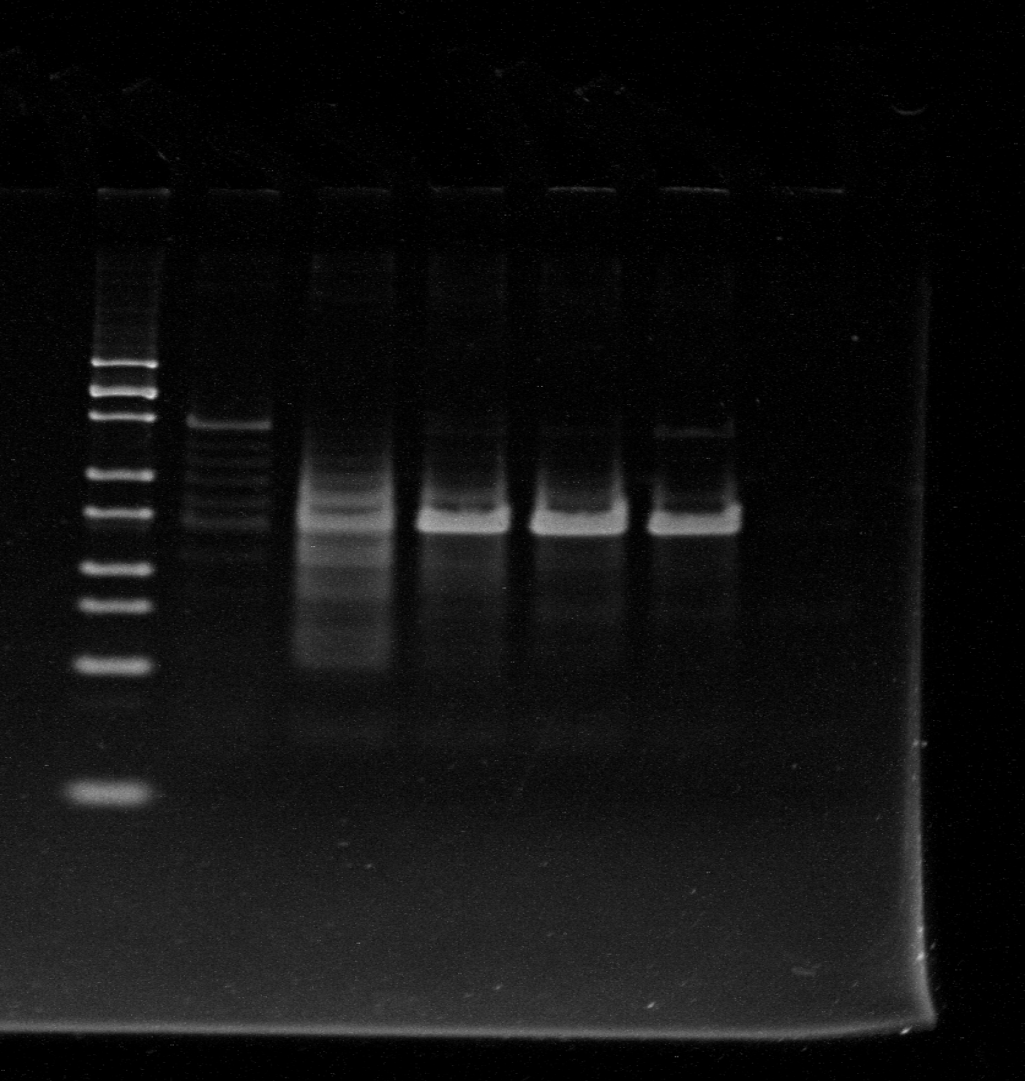
**

**RPA Nfo concentration optimisation results**

**
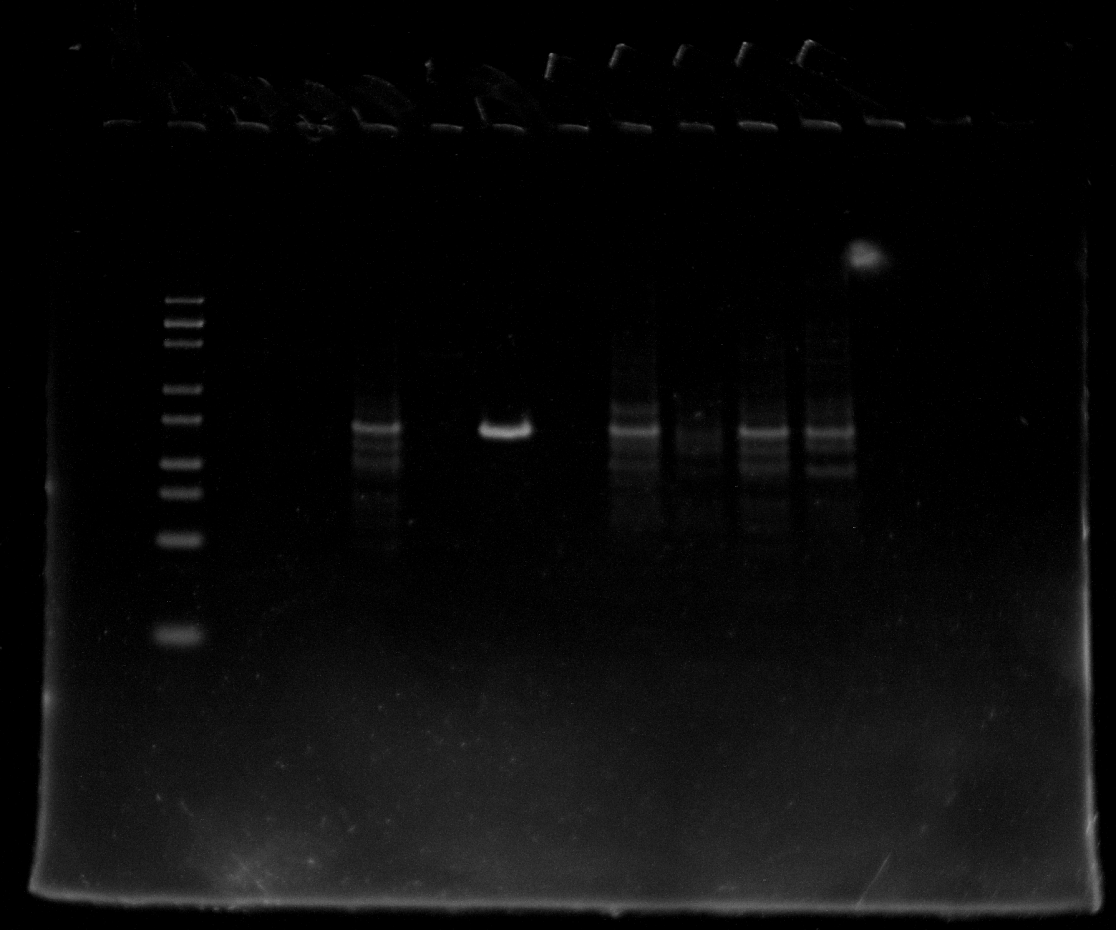
**

**qPCR primer concentration optimization results**

**
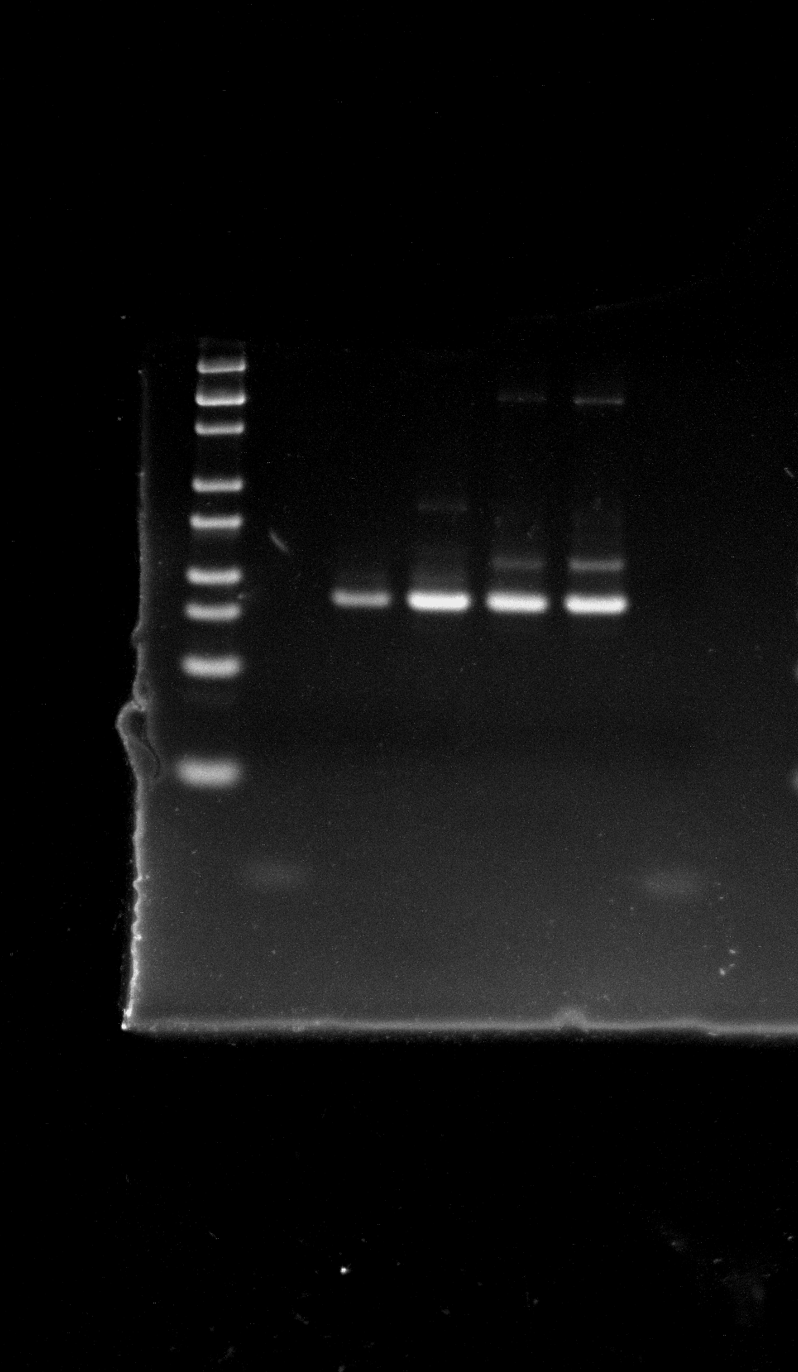
**

**qPCR probe concentration optimization results**

**
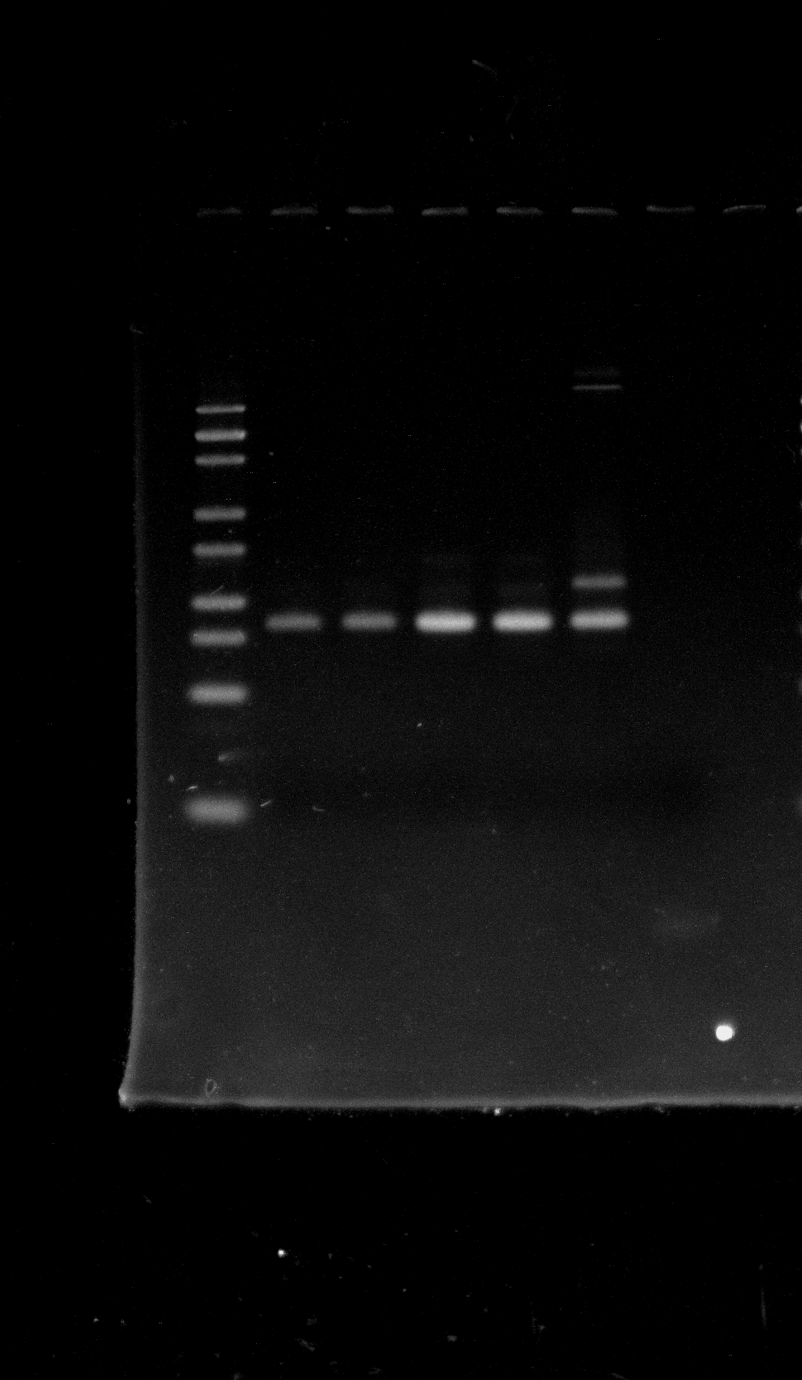
**

**qPCR annealing temperature optimization results**

**
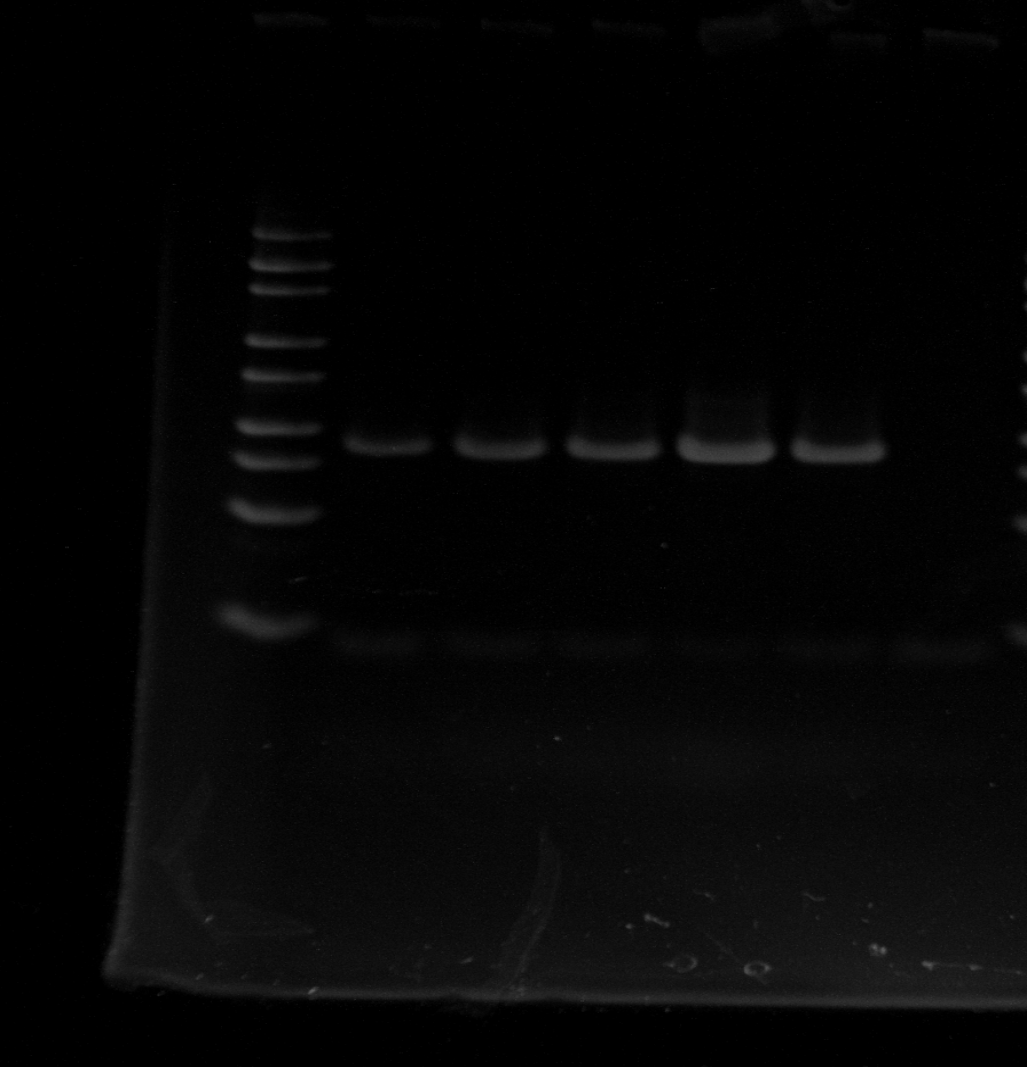
**

**HDA sensitivity test results graph**

**
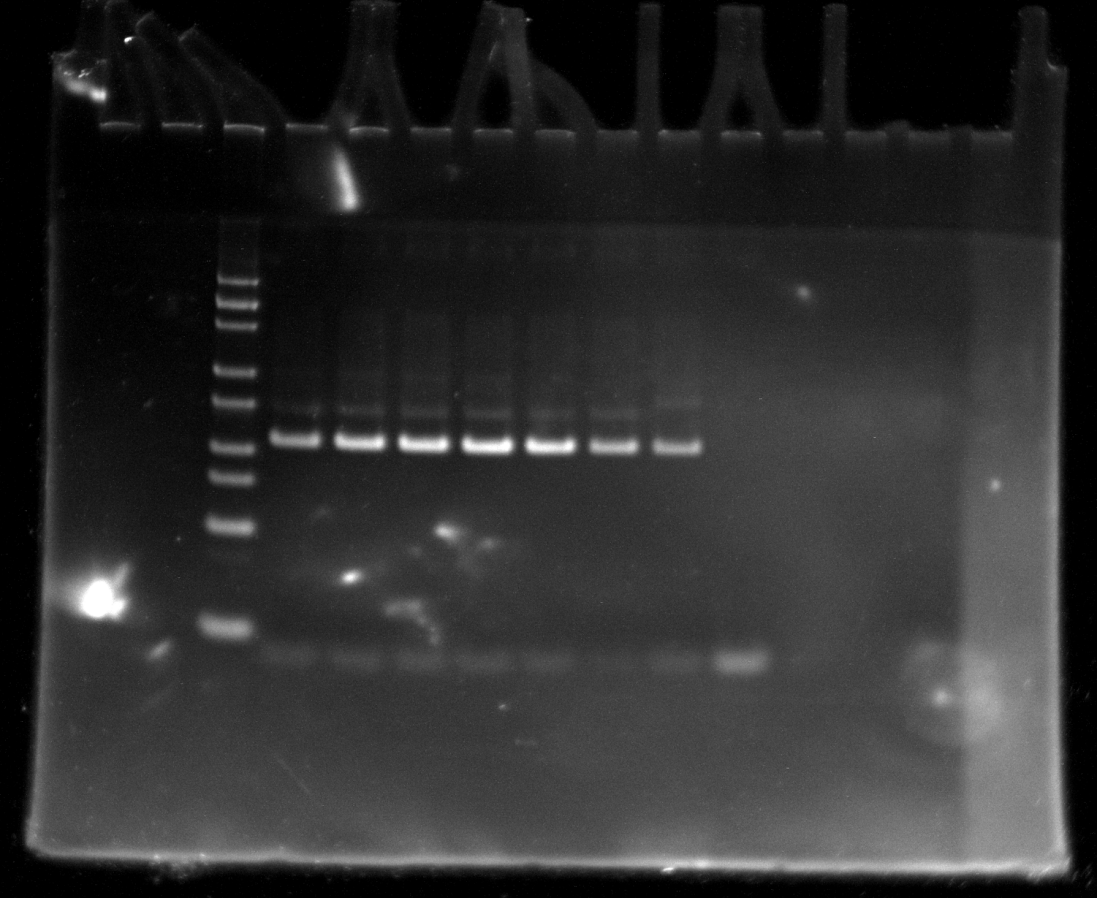
**

**RPA** **sensitivity test results graph**

**
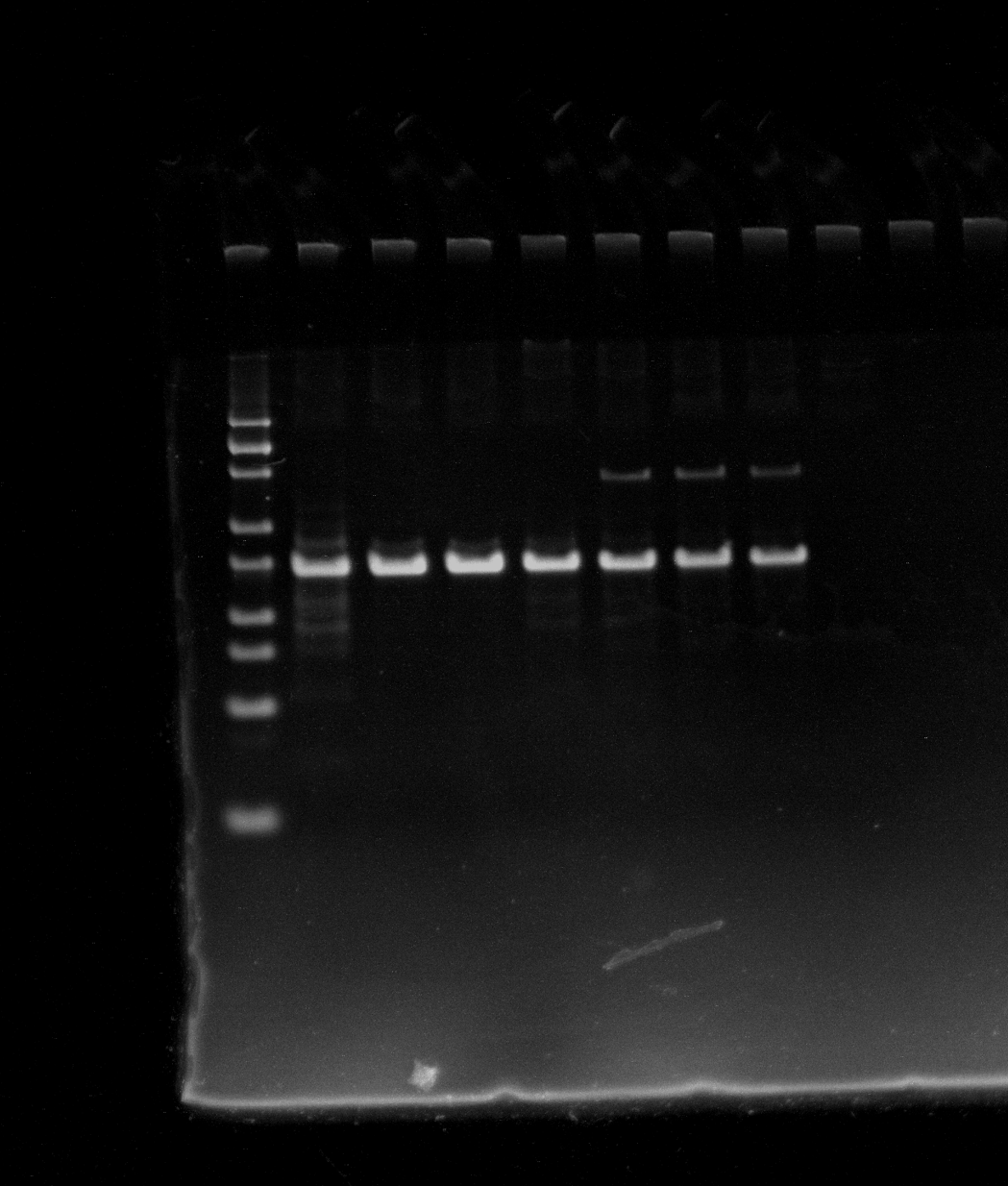
**

**qPCR sensitivity test results graph**

**
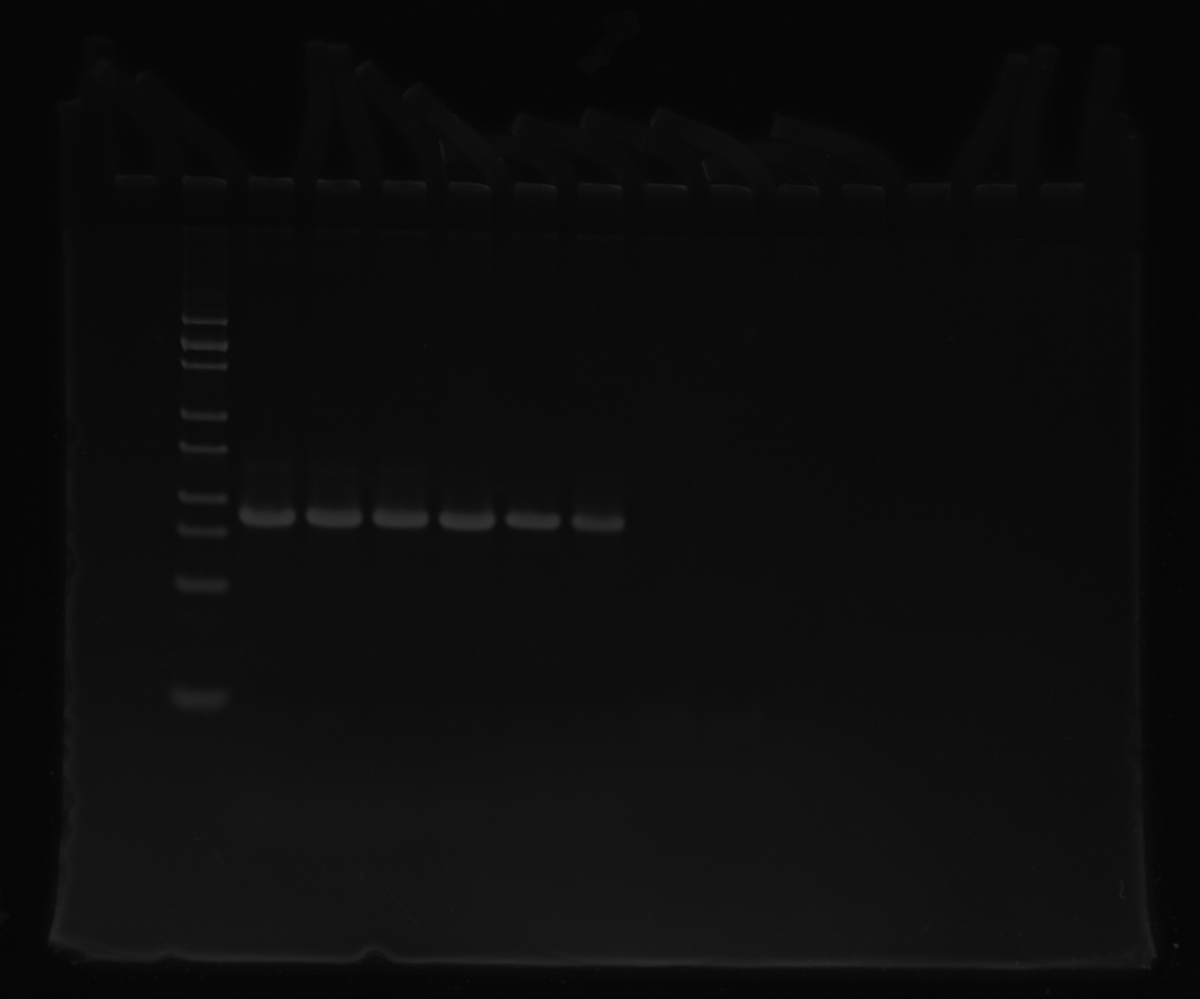
**

**HDA specificity test results graph**

**
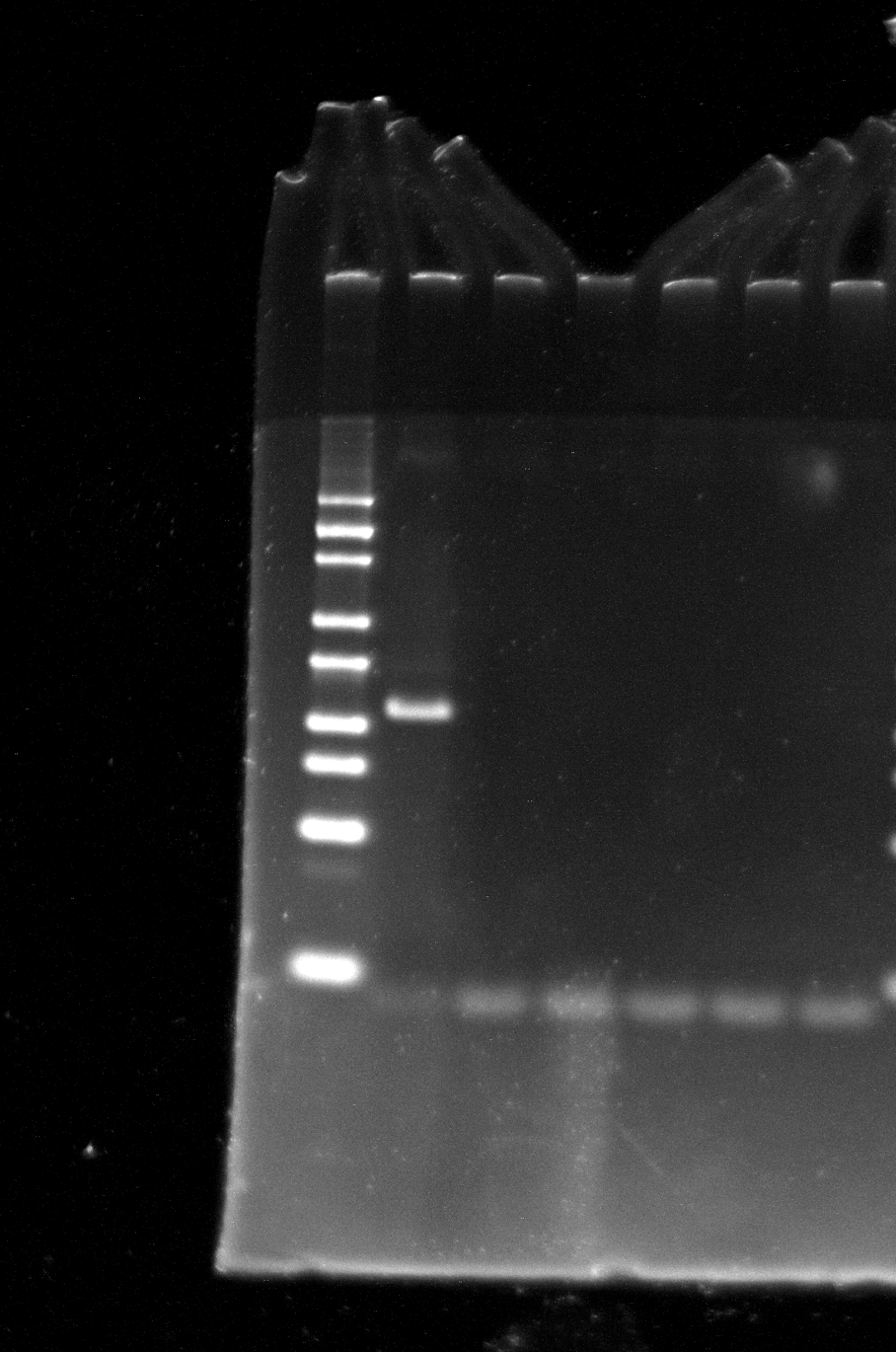
**

**RPA specificity test results graph**

**
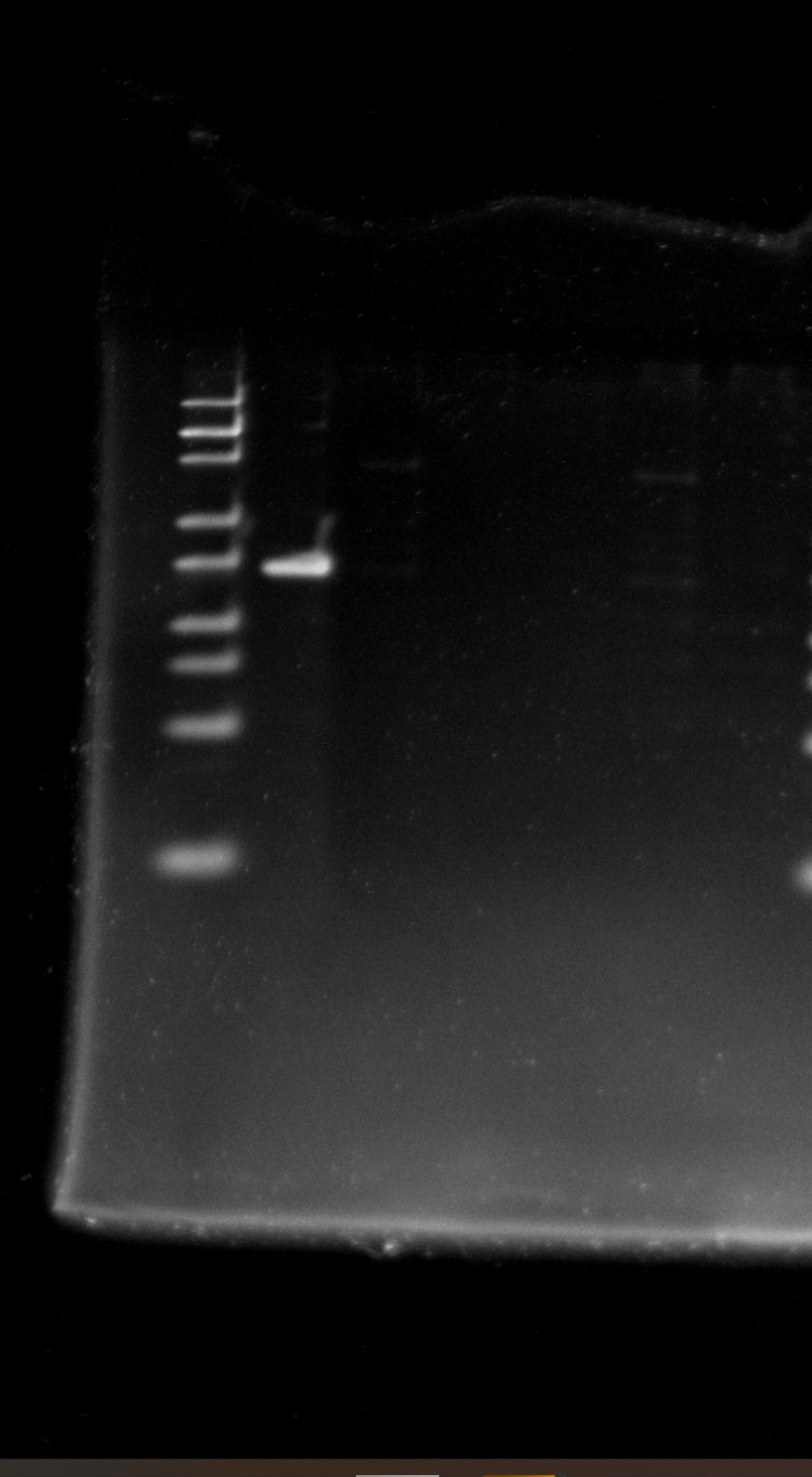
**

**qPCR specificity test results graph
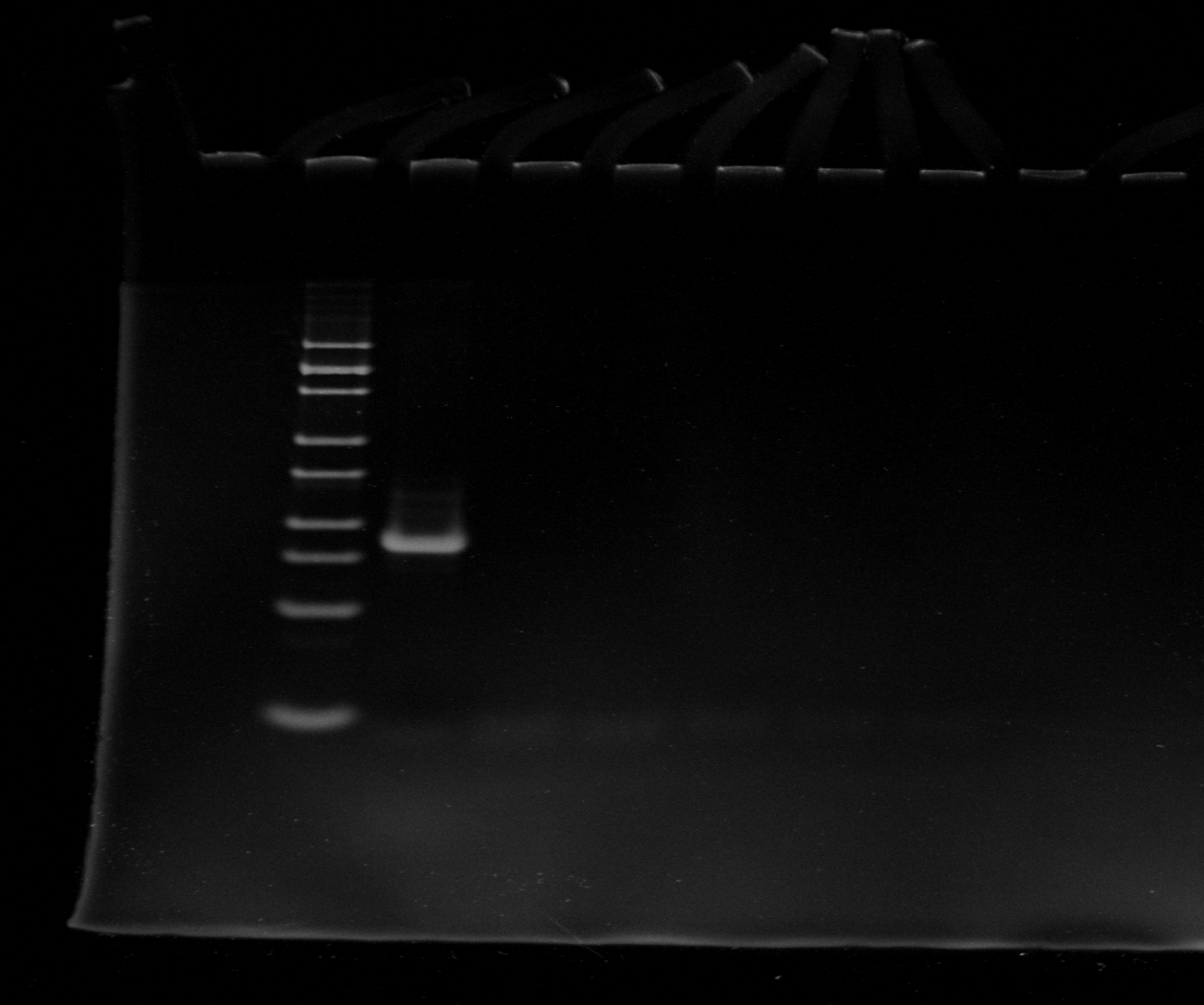
**
