# Supplementary material for: Detection of monkeypox virus using helicase dependent amplification and recombinase polymerase amplification combined with lateral flow test
Source: Virol J. 2023 Nov 23;20:274. doi: 10.1186/s12985-023-02223-8 (PMC10668421; doi:10.1186/s12985-023-02223-8)
Supplement: Supplementary file 2 — Supplementary Material 2 [file 12985_2023_2223_MOESM2_ESM.docx]

**Primer screening map for HDA-LFT:**


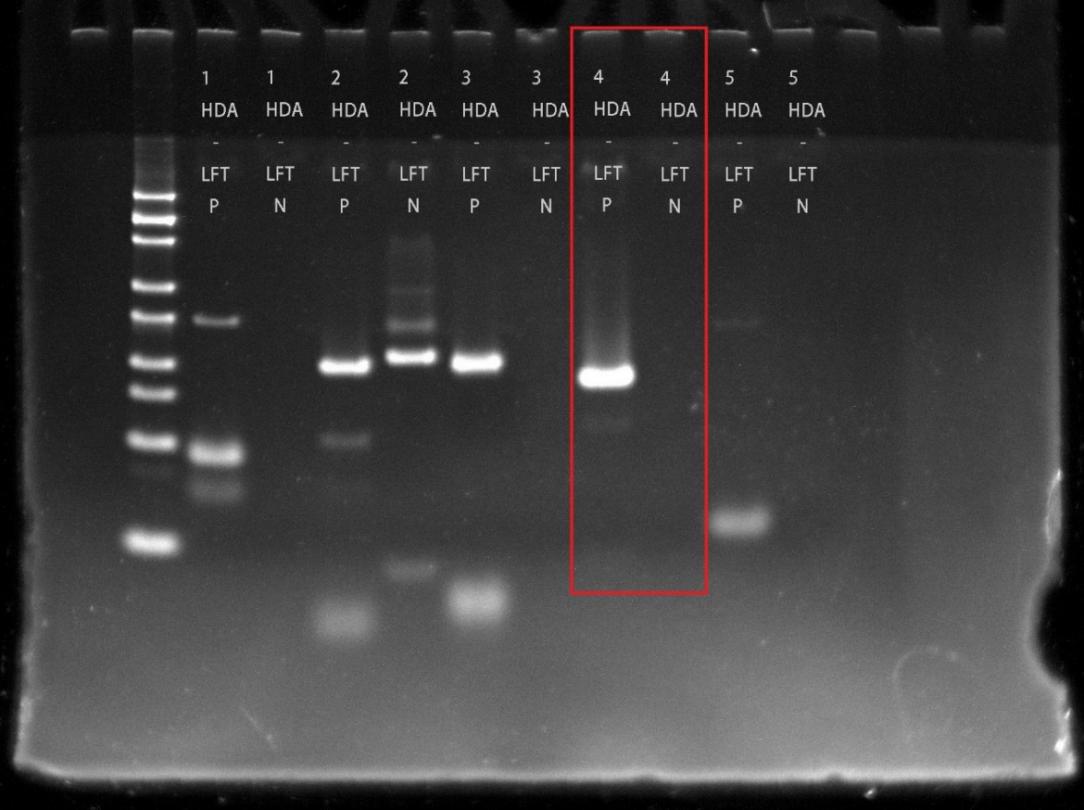


**Primer screening map for RPA-LFT:**


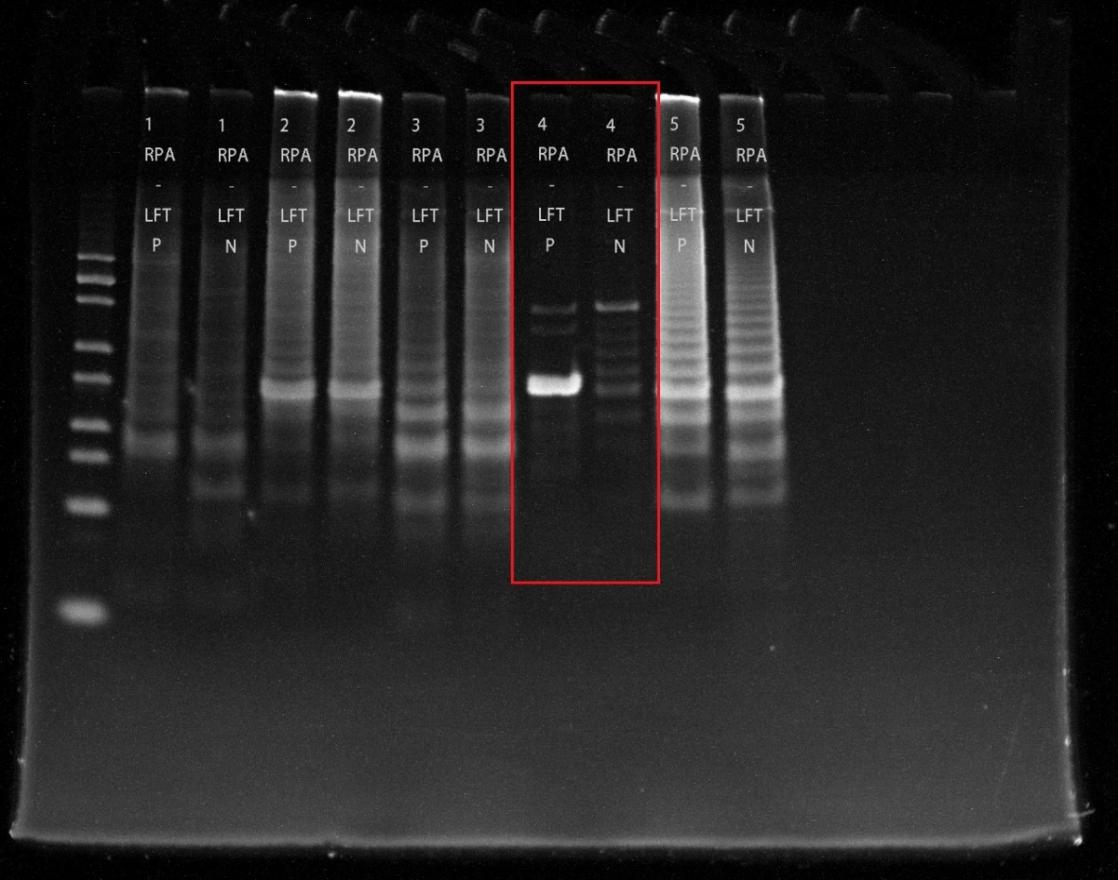


**Primer screening map for qPCR:**


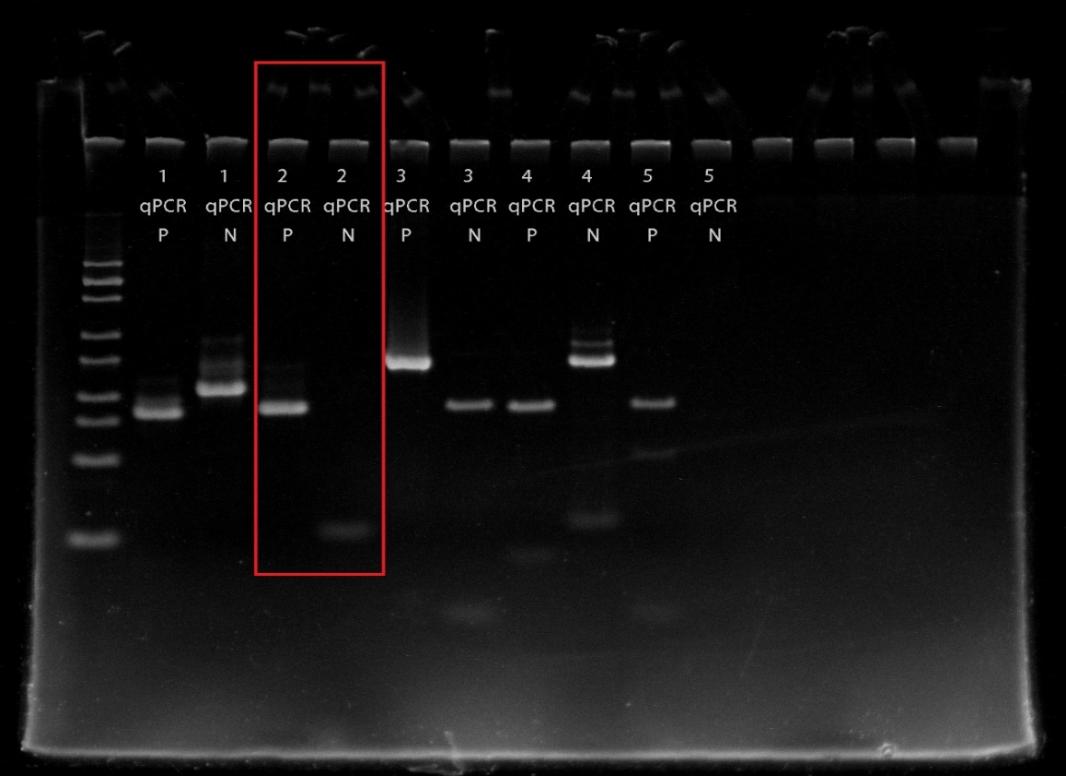


**HDA reaction time optimisation results**

**
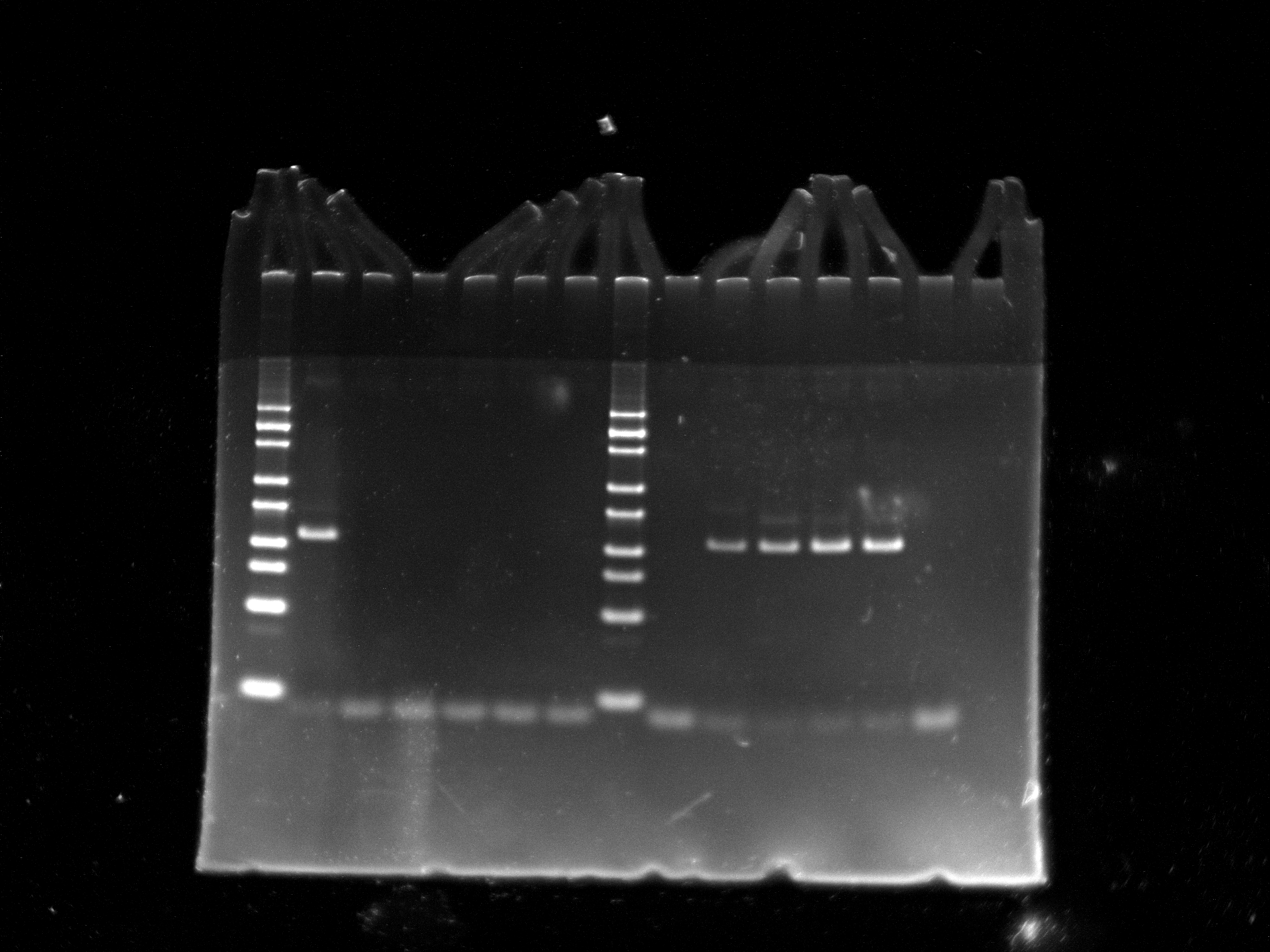
**

**HDA reaction temperature optimisation results**

**
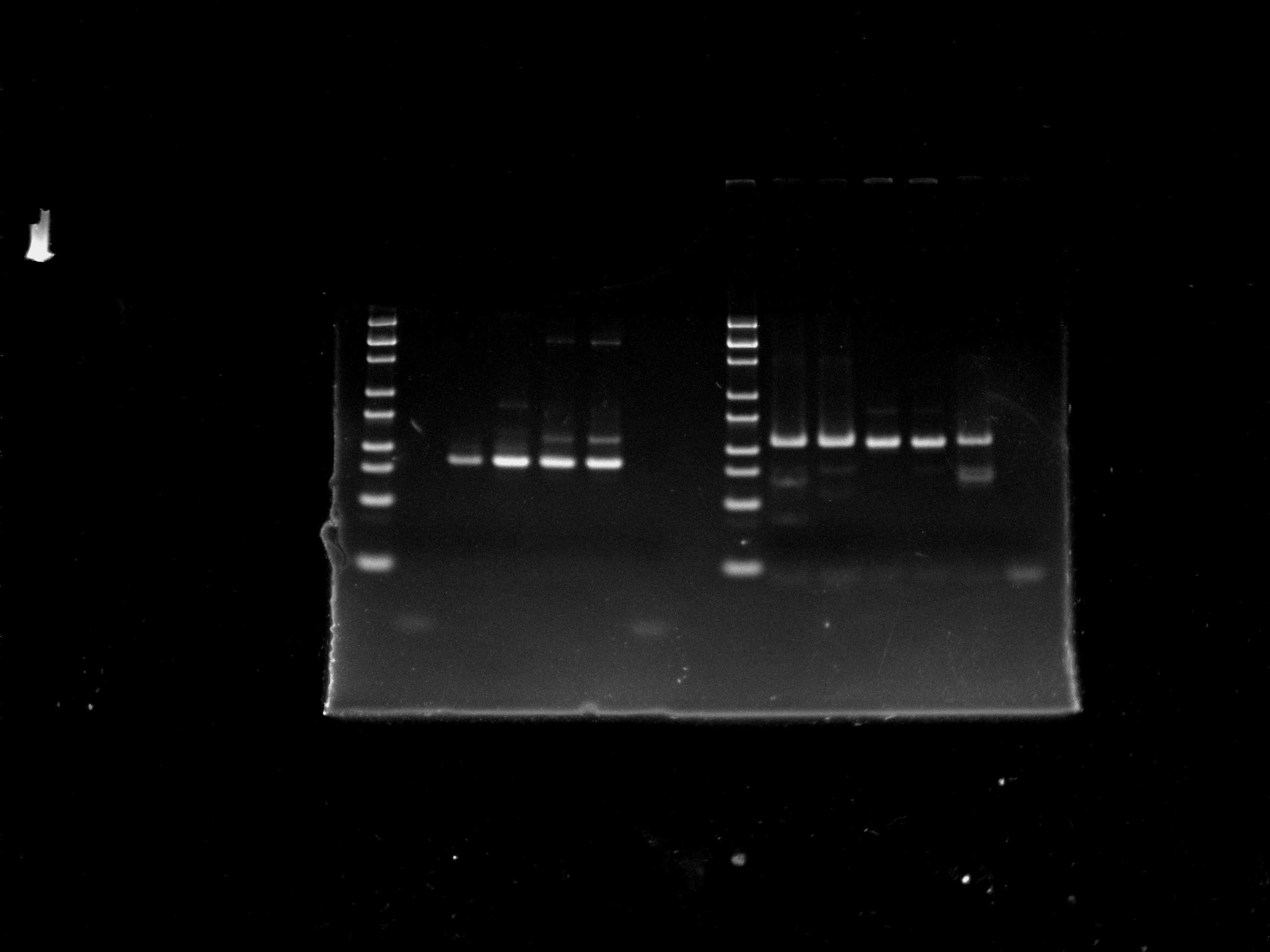
**

**HDA primer concentration optimization results**

**
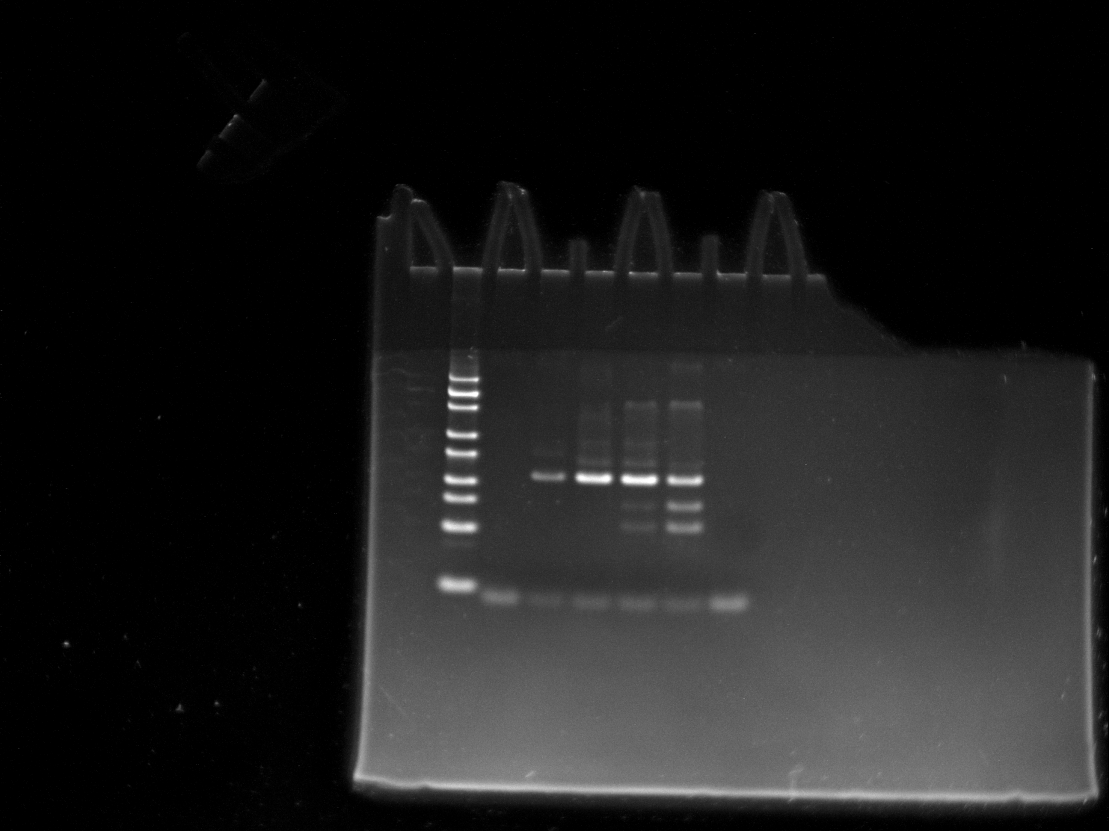
**

**HDA dNTPs concentration optimization results**

**
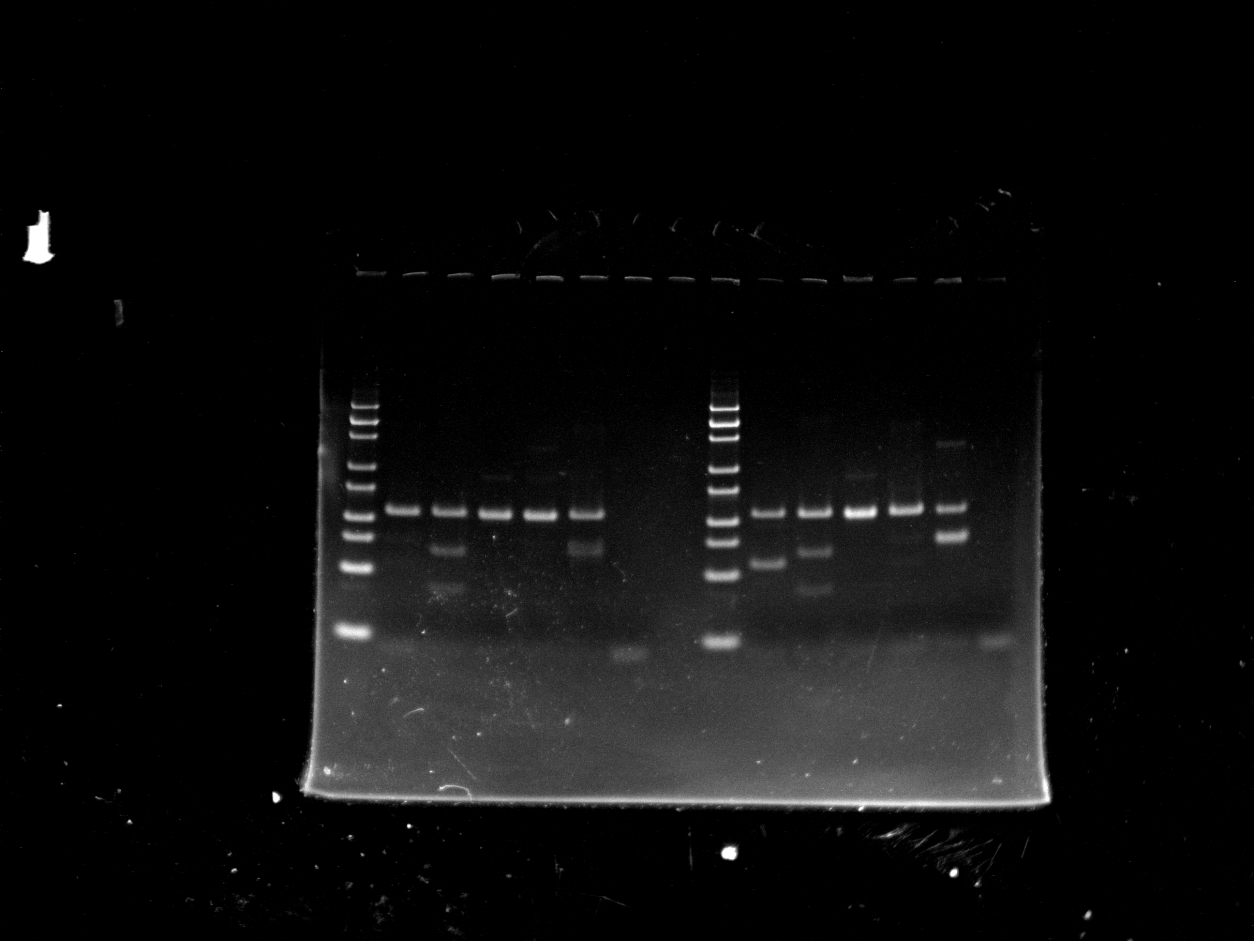
**

**RPA reaction time optimisation results**

**
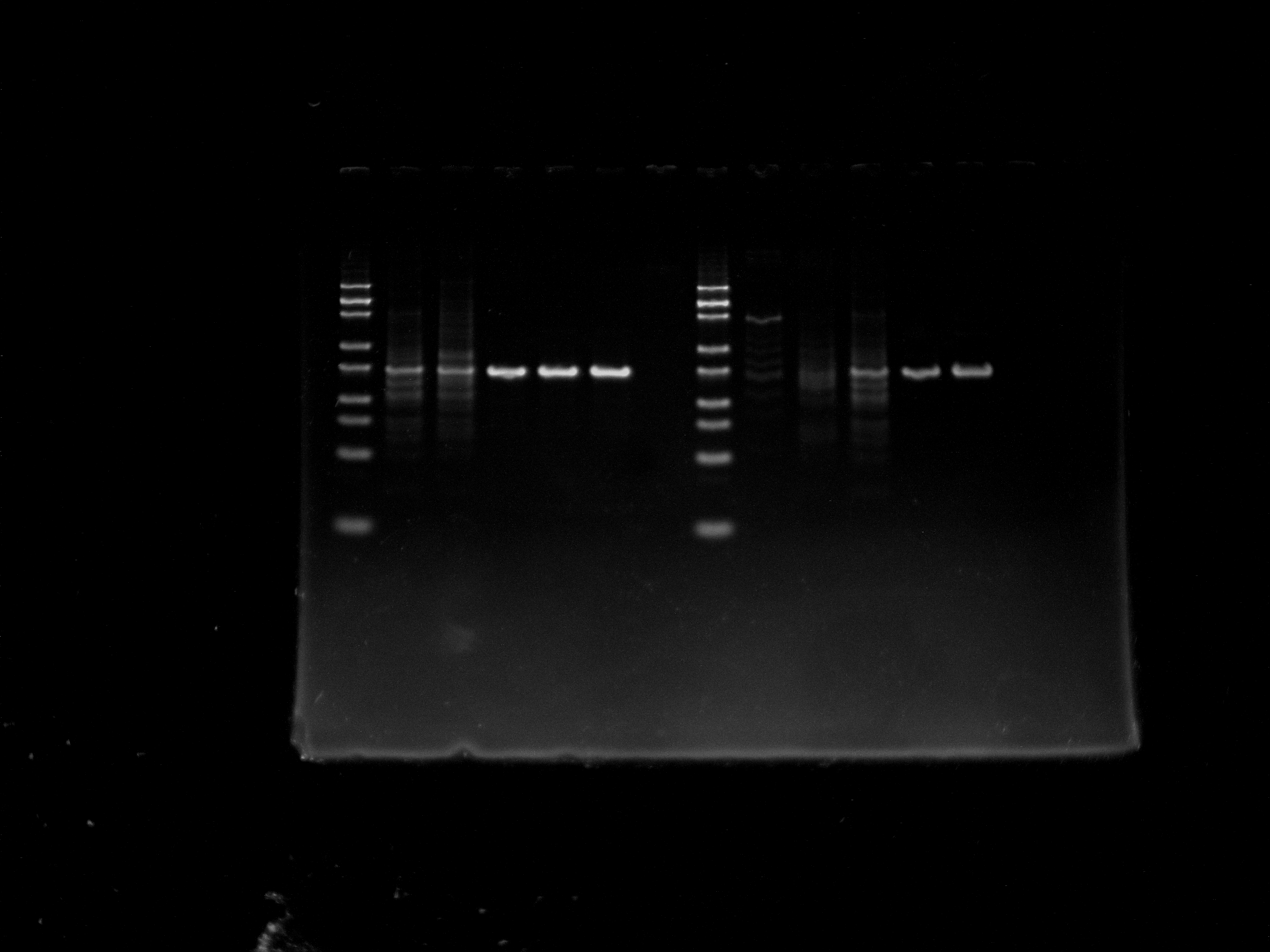
**

**RPA reaction temperature optimisation results**

**
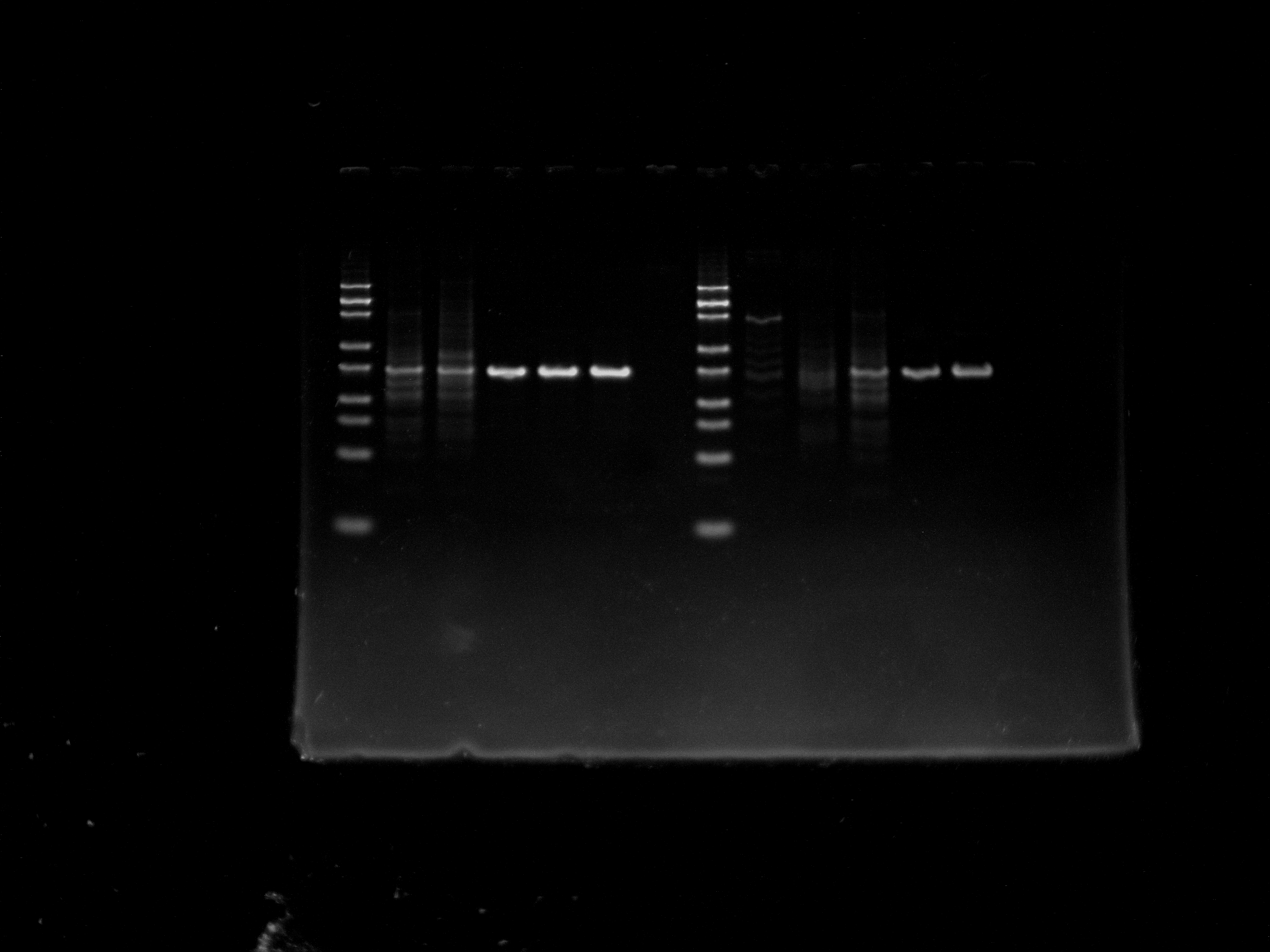
**

**RPA primer concentration optimization results**

**
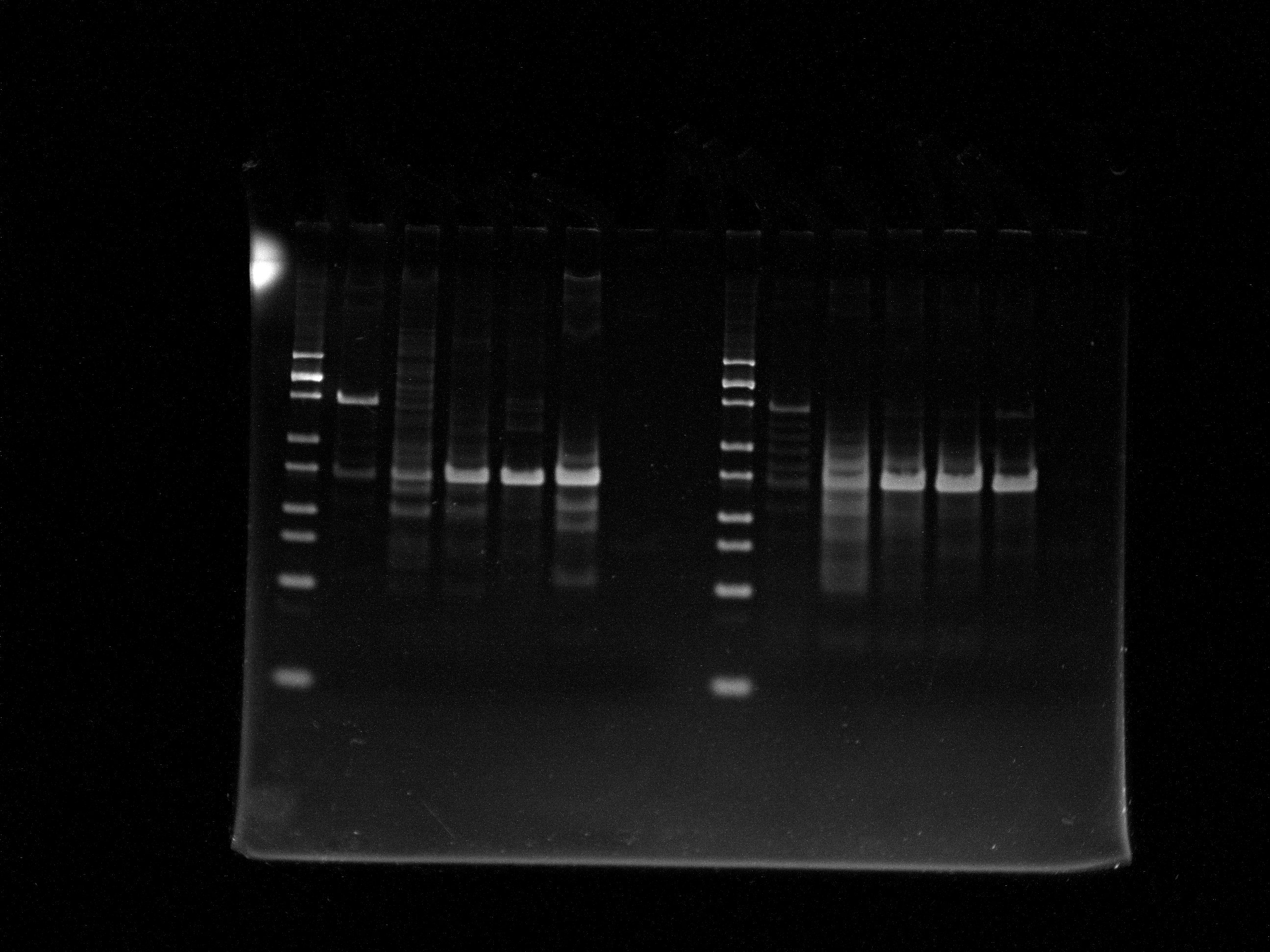
**

**RPA MgOAc concentration optimization results**

**
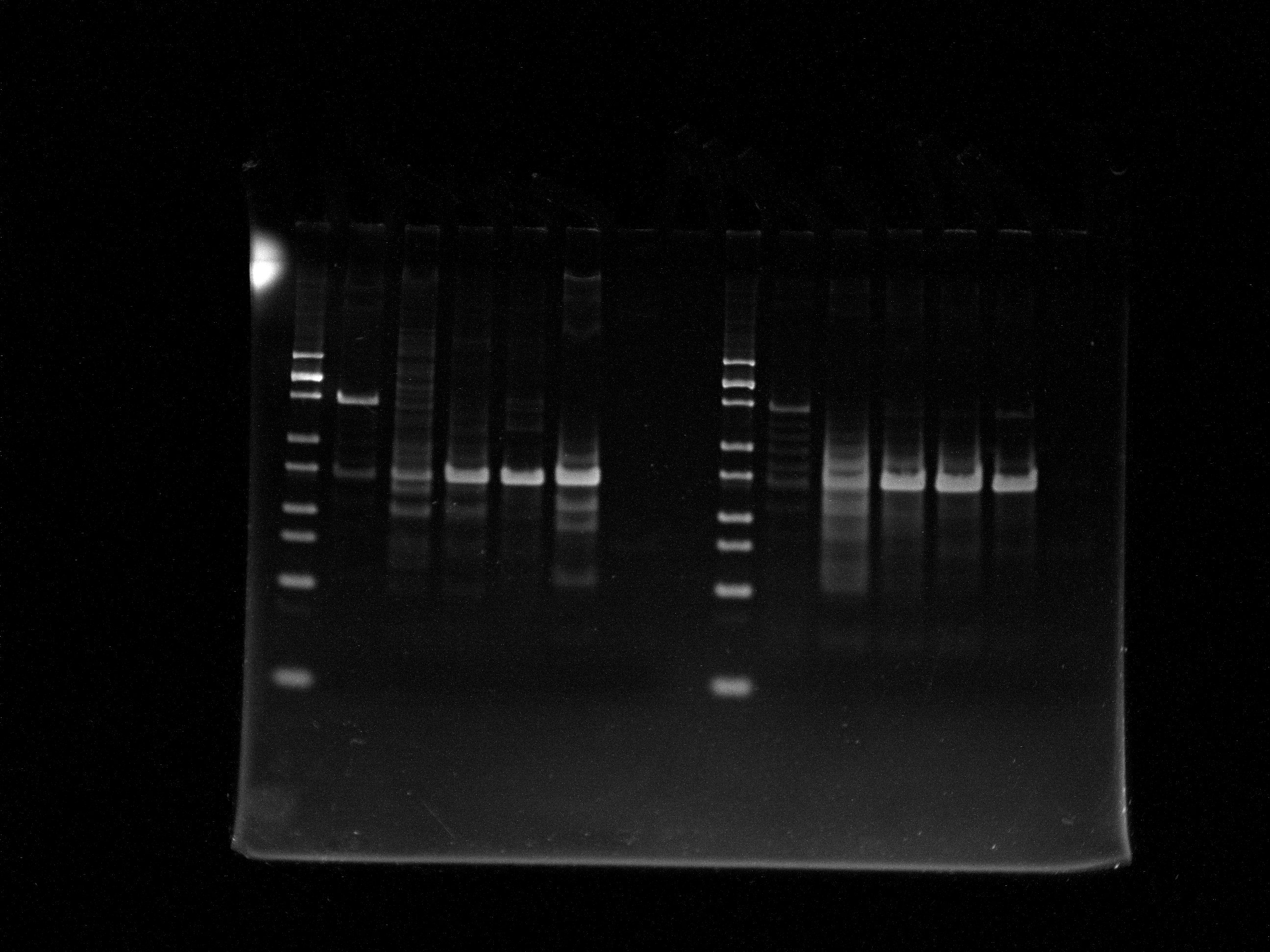
**

**RPA Nfo concentration optimisation results**

**
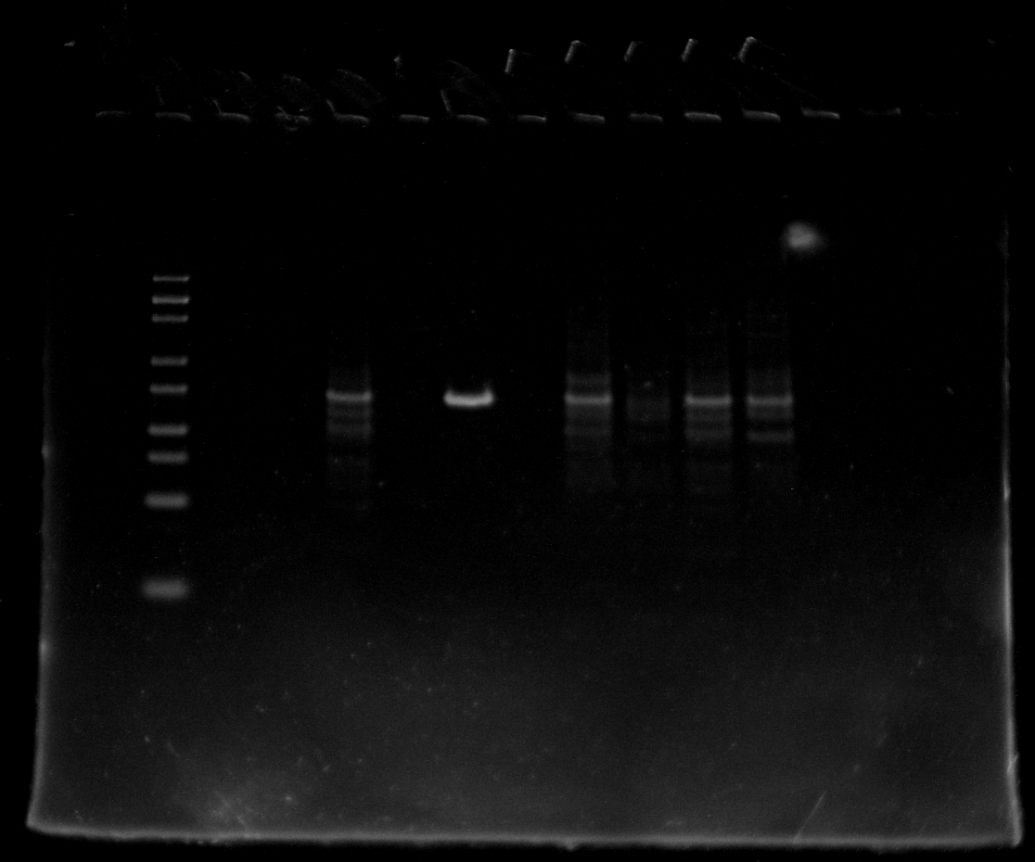
**

**qPCR primer concentration optimization results**

**
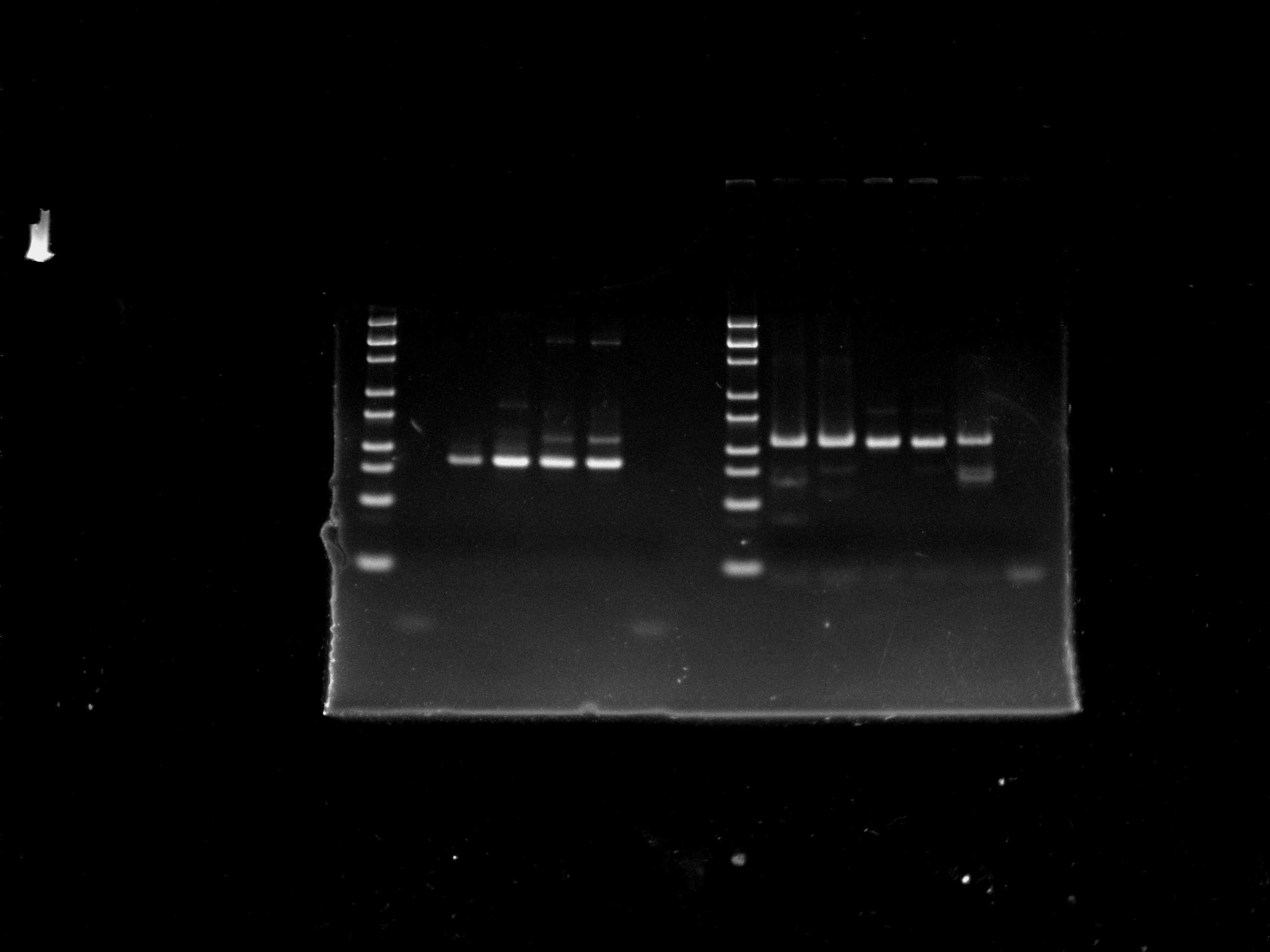
**

**qPCR probe concentration optimization results**

**
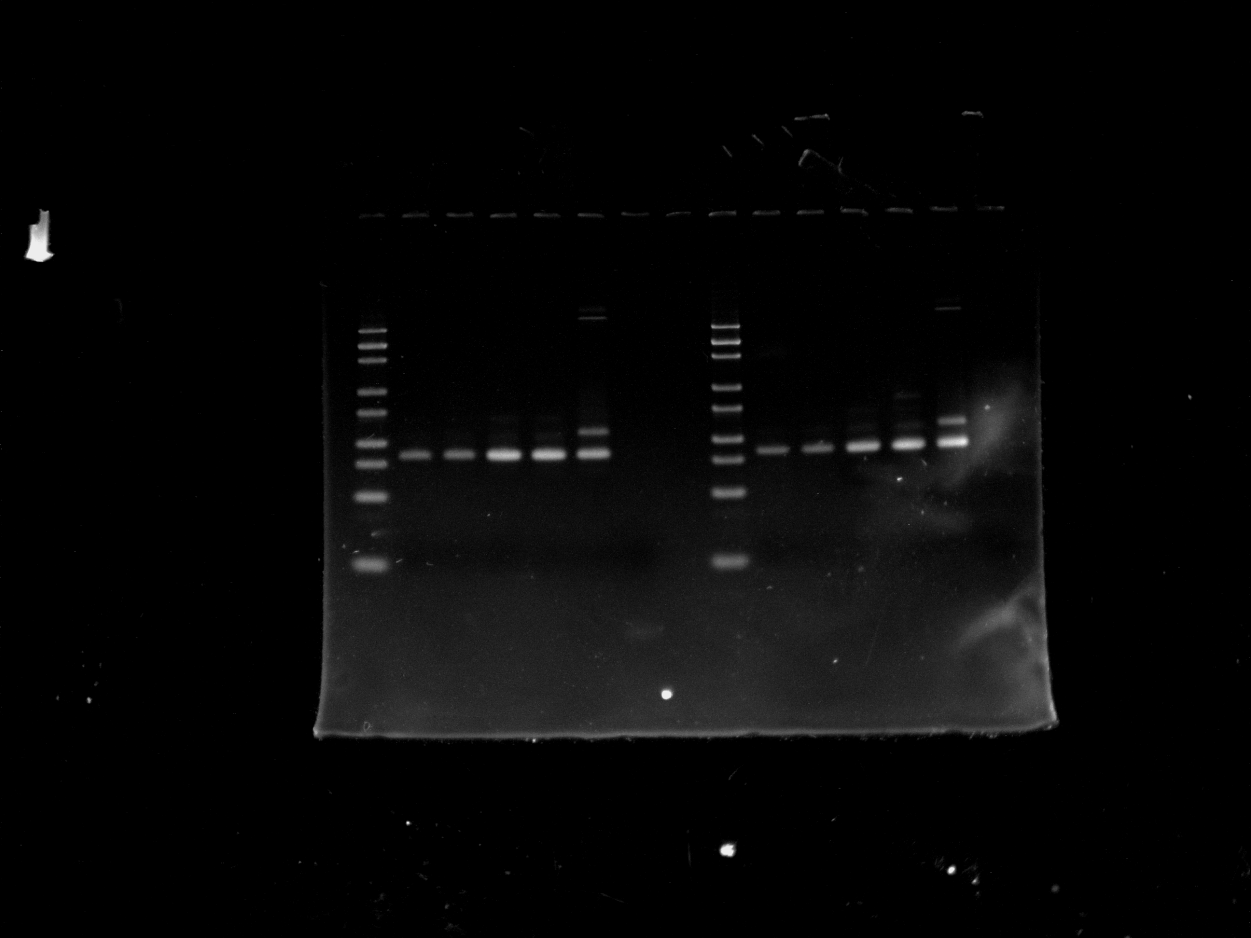
**

**qPCR annealing temperature optimization results**

**
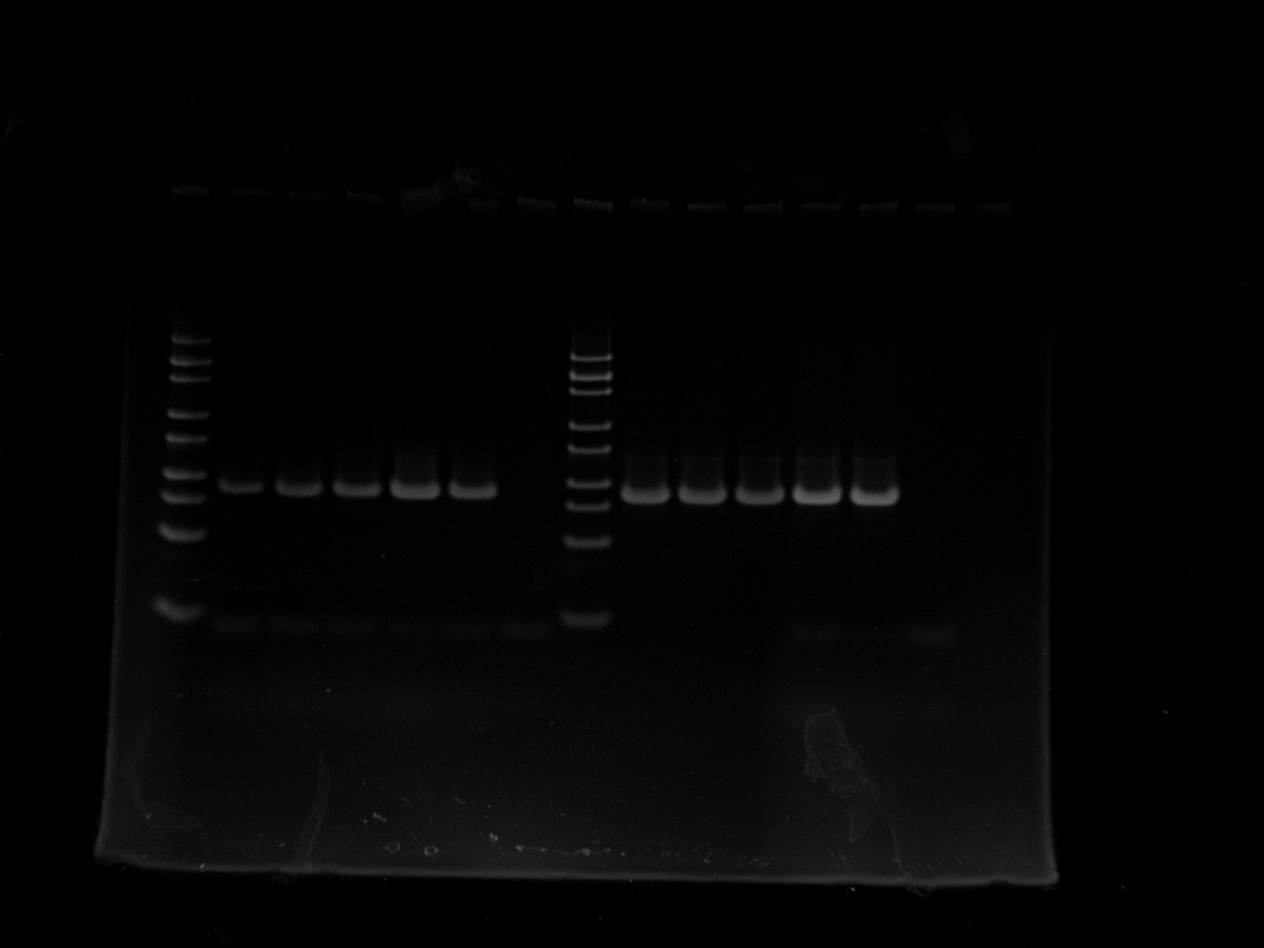
**

**HDA sensitivity test results graph**

**
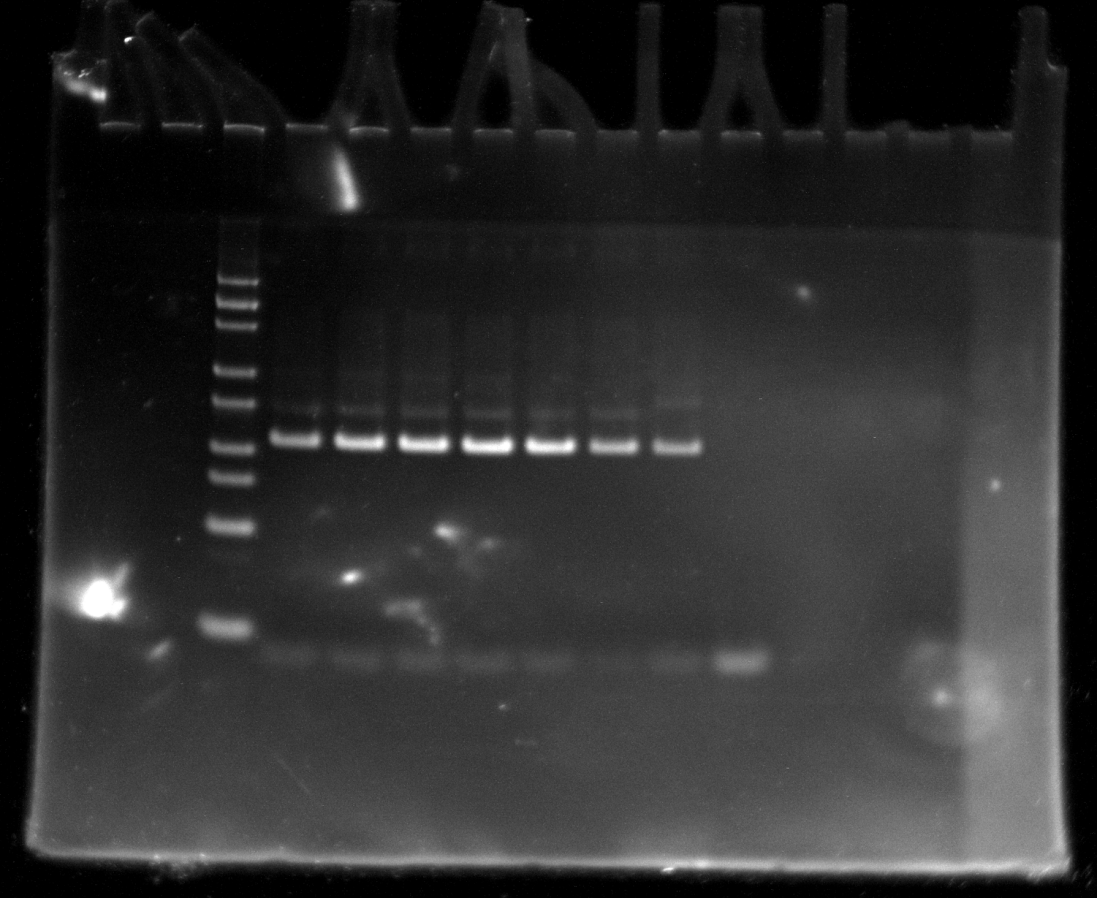
**

**RPA** **sensitivity test results graph**

**
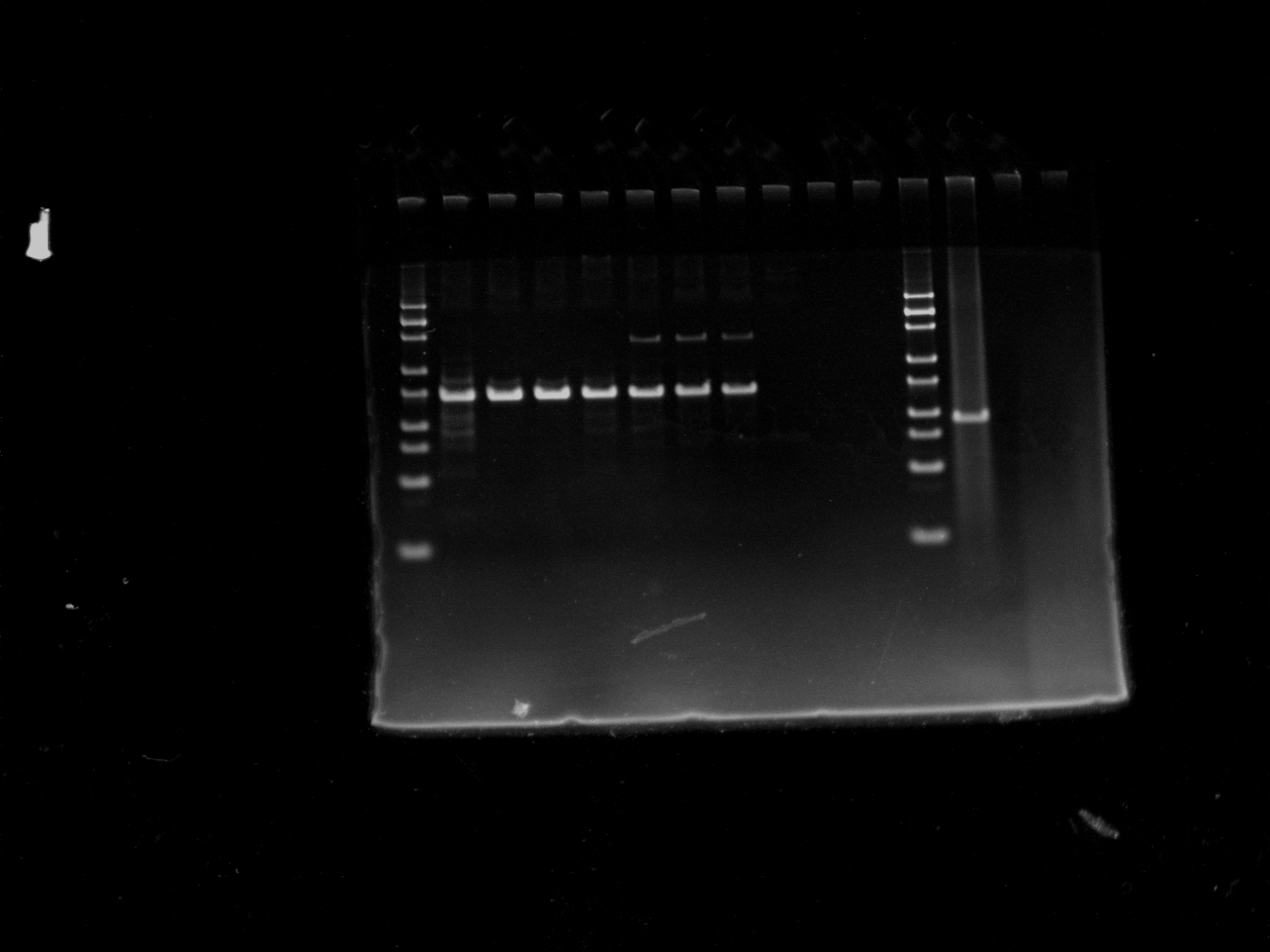
**

**qPCR sensitivity test results graph**

**
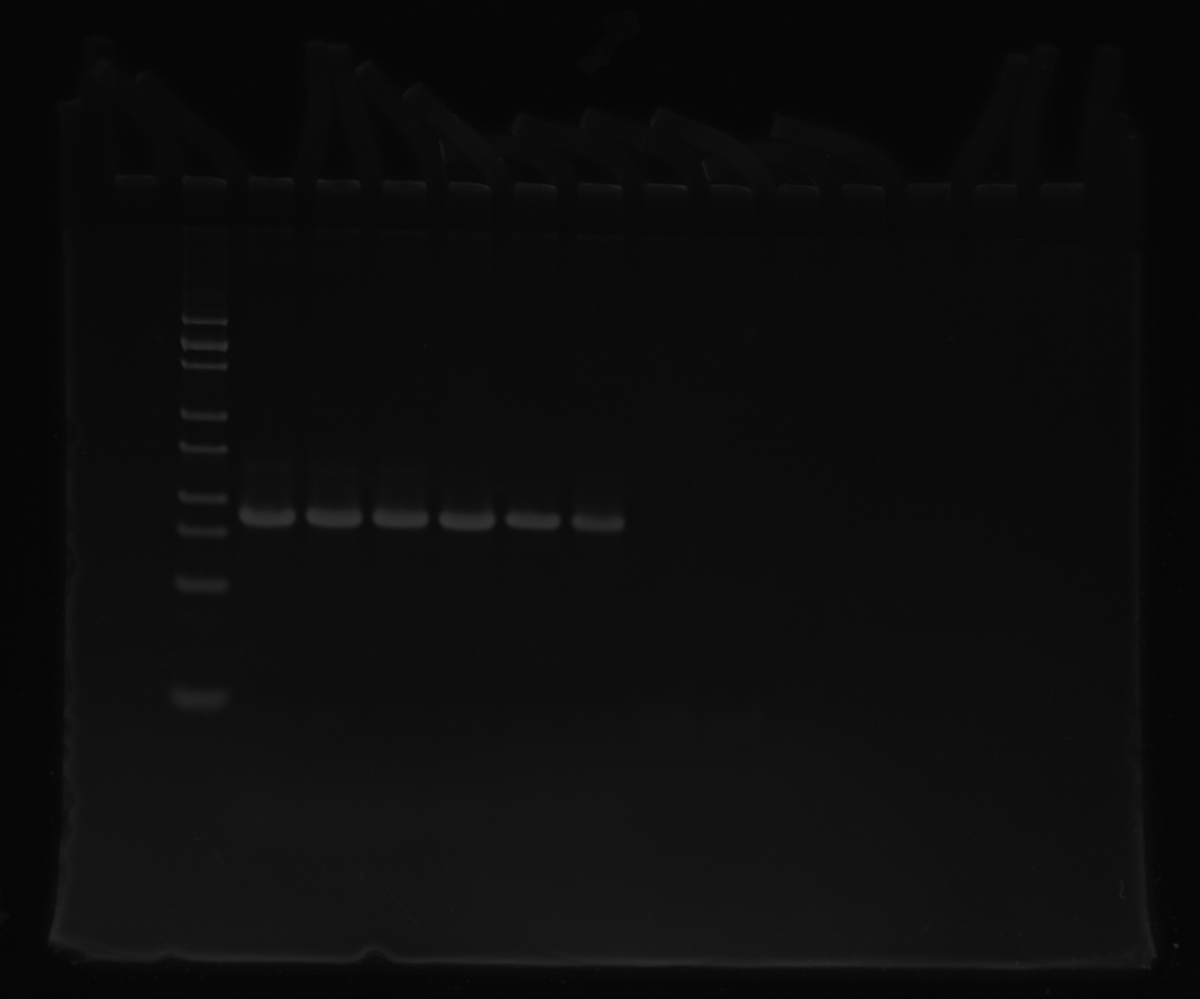
**

**HDA specificity test results graph**

**
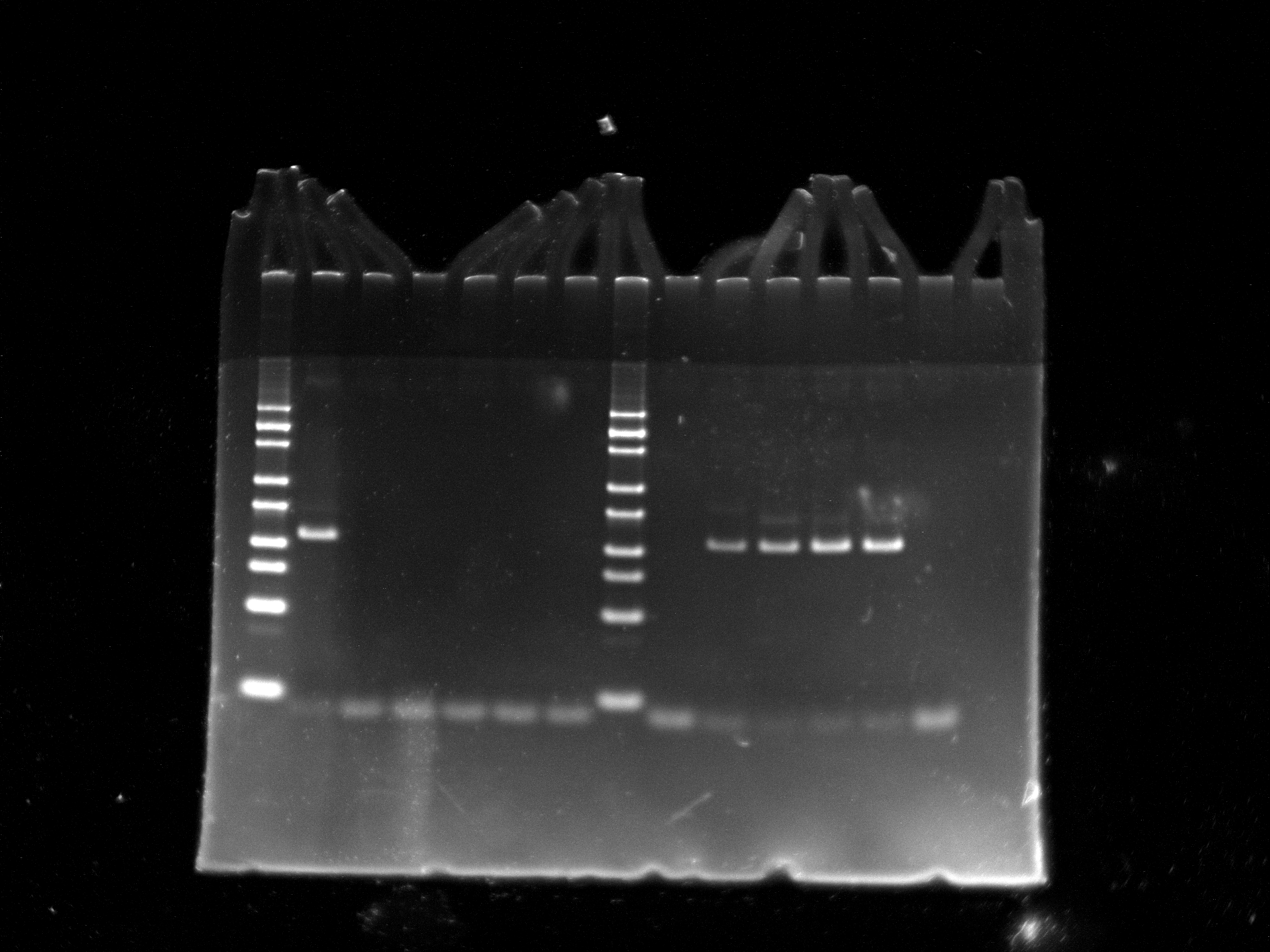
**

**RPA specificity test results graph**

**
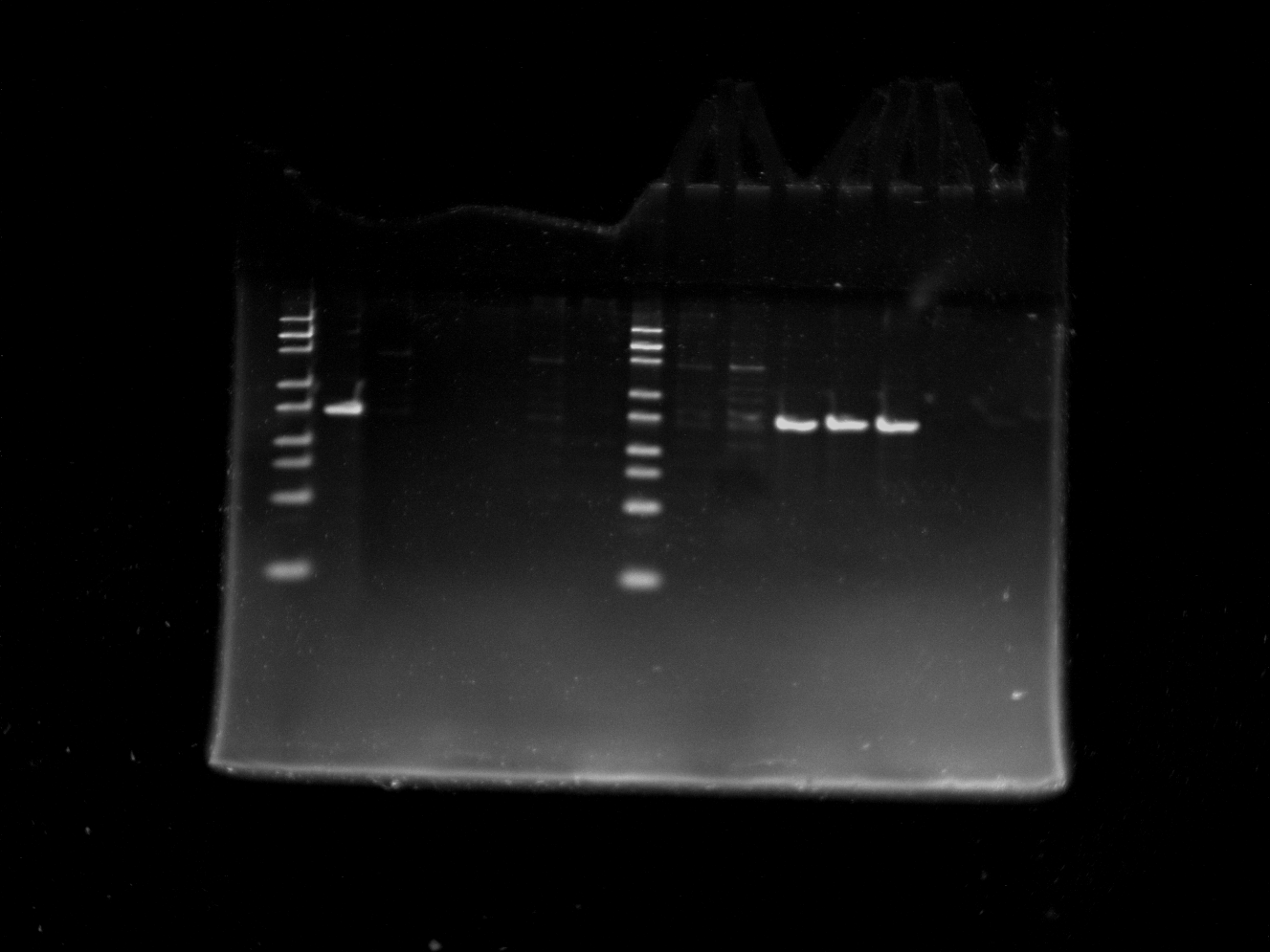
**

**qPCR specificity test results graph
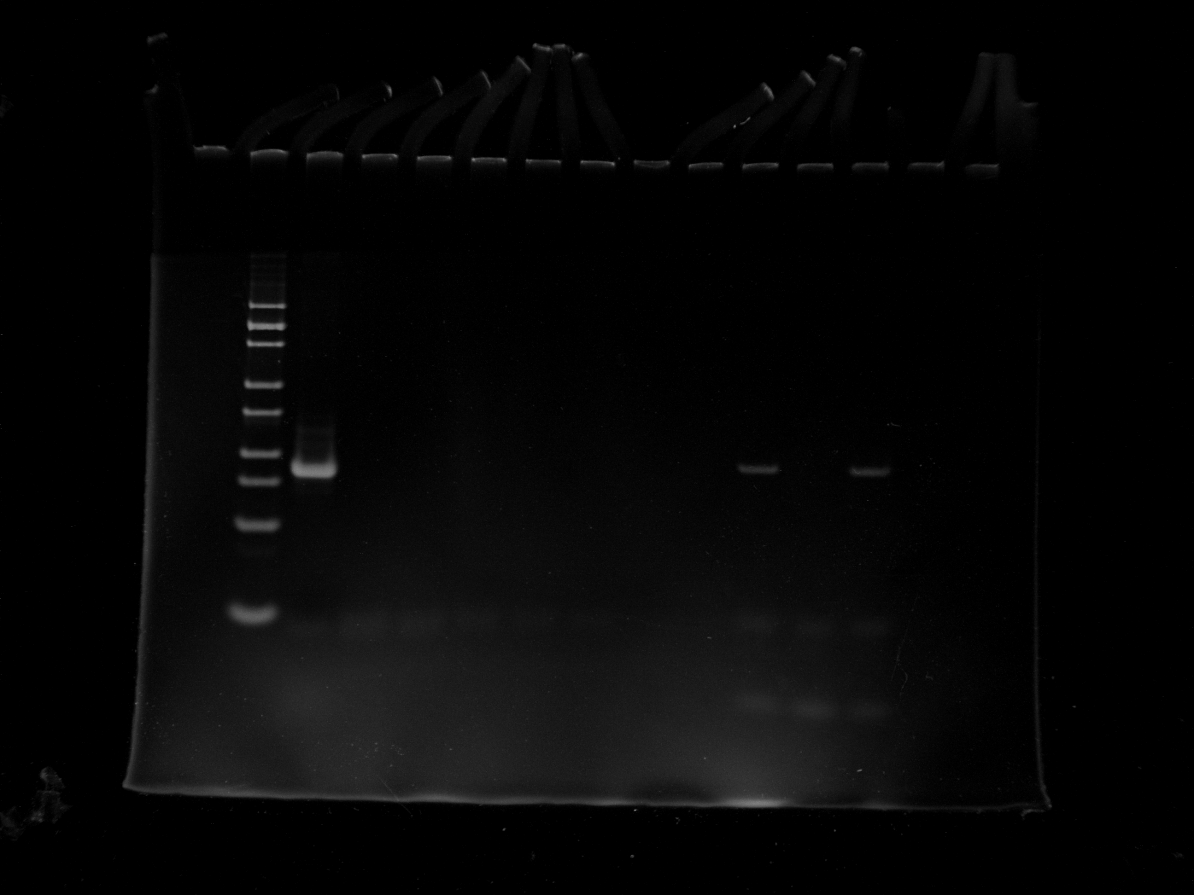
**
